# A Multilocus Integrative Framework to Reassess Species Boundaries Within the *Cystoseira* Sensu Stricto Complex (Fucales, Phaeophyceae)

**DOI:** 10.3390/plants15142237

**Published:** 2026-07-22

**Authors:** Sara D’Ambros Burchio, Alberto Pallavicini, Lucia Muggia, Ilaria Pagana, Giovanni Furnari, Samuele Greco, Elettra Chiarabelli, Fiorella Florian, Jose Valdazo, Ricardo Haroun, Anna Maria Mannino, Zahira Belattmania, Raquel Sanchez de Pedro, Polytimi Ioli Lardi, Maria Salomidi, Ljiljana Iveša, Claudio Battelli, Annalisa Falace

**Affiliations:** 1 Department of Life Sciences, University of Trieste, Via Licio Giorgieri 10, 34127 Trieste, Italy; 2 Consorzio Nazionale Interuniversitario per le Scienze del Mare (CoNISMa), Piazzale Flaminio 9, 00196 Roma, Italy; 3 National Biodiversity Future Center (NBFC), Piazza Marina 61, 90133 Palermo, Italy; 4 Department of Biological, Geological and Environmental Sciences, University of Catania, Via Empedocle 58, 95123 Catania, Italy; 5 Biodiversity & Conservation Research Group, ECOAQUA—University of Las Palmas de Gran Canaria, 35016 Las Palmas de Gran Canaria, Spain; 6 Pélagos Blue Restoration S.L., Centro de Innovación Marino Marítimo, Edificio Fundación Puerto Las Palmas, Muelle de Sta. Catalina, s/n, 35008 Las Palmas de Gran Canaria, Spain; 7 Department of Biological, Chemical and Pharmaceutical Sciences and Technologies, University of Palermo, Piazza Marina 61, 90127 Palermo, Italy; 8 Phycology, Blue Biodiversity and Biotechnology RU, Laboratory of Plant Biotechnology, Ecology and Ecosystem Valorization—CNRST Labeled Research Unit N.10, Faculty of Sciences, University Chouaib Doukkali, El Jadida 24000, Morocco; 9 Department de Botánica y Fisiología Vegetal, Campus de Teatinos, Universidad de Málaga, Andalucía Tech, 29010 Málaga, Spain; 10 Hellenic Centre of Marine Research (HCMR), Institute of Oceanography (IO), 19013 Anavyssos, Attica, Greece; 11 Ruđer Bošković Institute, Center for Marine Research, 52210 Rovinj, Croatia; 12 Slovenian Museum of Natural History, Prešernova Cesta 20, 1000 Ljubljana, Slovenia

**Keywords:** fucoid algae, haplotype networks, intraspecific diversity, phylogeny, species delimitation, taxonomy

## Abstract

*Cystoseira* sensu lato (s.l.) (Fucales, Phaeophyceae) form structurally complex marine forests along the warm-temperate coasts of the Mediterranean Sea and the eastern Atlantic Ocean. They are experiencing widespread decline, yet their conservation and restoration are hindered by taxonomic uncertainty. Within *Cystoseira* sensu stricto (s.s.), species boundaries among taxa traditionally referred to as *C. compressa*, *C. foeniculacea*, *C. humilis* (including the *C. canariensis* morphotype), and *C. pustulata* have long been debated due to the high plasticity and partial overlap of diagnostic morphological traits. Here we reassess species boundaries within the *Cystoseira* s.s. complex using an integrative approach combining morphology, ecology, and multilocus genetic data. We collected specimens from 20 sites spanning the Mediterranean–Atlantic distribution. Genomic DNA was extracted from 220 specimens and Sanger sequencing generated 243 new sequences (*ITS2*: *n* = 99; *cox1*: *n* = 88; *rbcL–rbcS*: *n* = 56). We inferred single-locus and concatenated phylogenies, reconstructed haplotype networks, and applied multiple single-locus species delimitation approaches to test the robustness of inferred boundaries. We consistently recovered two primary lineages corresponding to *C. foeniculacea* and a *C. compressa* complex, the latter including specimens historically identified as *C. pustulata* and the *C. humilis* with the *C. canariensis* morphotype. Within an integrative framework that prioritises cross-locus concordance and diagnosability, we recognise two species-level lineages in *Cystoseira* s.s. and treat the morpho-ecologically coherent entities within *C. compressa* at the variety rank. Accordingly, we propose the new status and combination *Cystoseira compressa* var. *pustulata* (Ercegović) D’Ambros Burchio & Falace, comb. et stat. nov.

## 1. Introduction

Brown macroalgae of the genus *Cystoseira* sensu lato (s.l.) (Fucales, Phaeophyceae) form extensive marine forests along the warm-temperate coasts of the Mediterranean Sea and the eastern Atlantic Ocean [1,2,3,4]. As ecosystem engineers [5], these canopy-forming assemblages create complex three-dimensional habitats that support high biodiversity and provide multiple ecosystem services [6,7,8]. In recent decades, *Cystoseira* s.l. forests have experienced widespread decline across much of their range [9,10,11,12], driven by multiple anthropogenic stressors, including eutrophication, habitat degradation, and ocean warming [9,13,14]. Within this declining group, taxonomic uncertainty remains a major obstacle to accurate biodiversity assessment and effective conservation planning, particularly for morphologically complex lineages.

Accurate species identification within *Cystoseira* s.l. is notoriously challenging due to pronounced phenotypic plasticity [15,16,17], ongoing or recent speciation [18,19], and frequent hybridisation [19,20]. Nevertheless, robust taxonomy is a prerequisite for detecting shifts in species distributions, documenting local extinctions, and defining conservation priorities, particularly for habitat-forming taxa undergoing rapid ecological change, such as *Cystoseira* s.l. [21,22,23]. The limitations of morphology-based taxonomy have therefore prompted increasing use of molecular approaches to delineate species boundaries and clarify evolutionary relationships within the group [1,17,24,25,26,27,28,29,30].

While molecular data have greatly improved taxonomic resolution in *Cystoseira* s.l., their application has not always been consistent across studies, and species boundaries remain unstable in several complexes. Molecular phylogenetic studies have shown that *Cystoseira* s.l., as traditionally circumscribed, represents a polyphyletic assemblage [17]. Based on multilocus evidence, Draisma et al. [17] proposed a major taxonomic reorganisation of the family Sargassaceae, reinstating the genera *Stephanocystis* Trevisan, *Polycladia* Montagne, and *Sirophysalis* Kützing for species in the North Pacific, western Indian Ocean, and Indo-Pacific regions, respectively. Although these authors suggested that the European representatives of *Cystoseira* should also be segregated into multiple genera, they did not formally propose new combinations. Subsequent phylogenetic analyses focusing on European taxa refined this framework. Orellana et al. [1] restricted *Cystoseira* sensu stricto (s.s.) to a single well-supported clade and resurrected the historical genera *Carpodesmia* Greville and *Treptacantha* Kützing for the remaining Mediterranean-Atlantic species. Shortly thereafter, Molinari and Guiry [26] clarified that the correct names for these genera are *Ericaria* Stackhouse and *Gongolaria* Boehmer, which have nomenclatural priority over *Carpodesmia* and *Treptacantha*, respectively. More recently, Neiva et al. [27] corroborated this generic framework using *cox1* barcoding data from the northeastern Atlantic and western Mediterranean, while highlighting several unresolved species complexes.

Despite these advances, resolution of species complexes requires an integrative taxonomic framework that explicitly combines multiple lines of evidence. Different studies have applied heterogeneous analytical strategies, with some combining genetic analyses and morphology [1,31,32], while others have relied exclusively on molecular data. Furthermore, marker choice has varied according to the taxonomic scale investigated. For example, the plastid-encoded *psbA* gene has been used primarily to infer higher-level relationships, whereas mitochondrial markers such as *mt23S* and the *mt23S–tRNA^Val* spacer have proved informative at the genus level [1,17]. At the species level, most studies have relied on the mitochondrial *cox1* gene [1,25,27,32]. In other Phaeophyceae, however, plastid (*rbcL–rbcS*) and nuclear (*ITS2*) markers have been shown to provide complementary resolution for species delimitation and intraspecific analyses [33,34]. This heterogeneity in marker choice and analytical frameworks has hampered comparability among studies, highlighting the need for a standardised multilocus approach.

Among the taxa currently included within *Cystoseira* s.s., we focus here on four entities presently treated at species rank: *C. compressa* (Esper) Gerloff & Nizamuddin, *C. foeniculacea* (Linnaeus) Greville, *C. humilis* Schousboe ex Kützing (with the morphology of *C. canariensis* Sauvageau), and *C. pustulata* (Ercegović) Neiva & Serrão. Their taxonomic history is complicated by pronounced morphological plasticity and partially overlapping distributions, which have led to frequent misidentifications and unstable classifications [35,36,37,38]. Traditional diagnostic traits, including branch compression, aerocyst development, and cryptostomata morphology, are highly plastic and strongly influenced by environmental conditions such as hydrodynamic exposure, light availability, and tidal regime, often obscuring species boundaries.

Consequently, multiple taxonomic revisions have produced conflicting interpretations and descriptions without fully resolving the status of these entities [16,35,38,39,40,41,42]. Neiva et al. [27] recently reassessed the group using *cox1*, supporting recognition of four taxa within *Cystoseira* s.s.; however, reliance on a single locus may be insufficient where shallow divergence, hybridisation, and morphological overlap coexist [20,43]. A multilocus approach, explicitly integrated with morphological and ecological traits, is therefore preferable to robustly delineate species boundaries and to test whether the currently recognised taxa represent independent evolutionary lineages.

To address these issues, we applied a multilocus phylogenetic analysis of three complementary DNA markers (*cox1, rbcL–rbcS*, *ITS2*) and integrated it with detailed morphological analyses and ecological data. In doing this we aim to: (i) reassess species boundaries within *Cystoseira* s.s., using congruent multilocus phylogenetic and species delimitation analyses; (ii) evaluate the taxonomic significance of morpho-ecological differentiation within the *C. compressa* complex; and (iii) provide a robust, reproducible reference framework to support biodiversity monitoring, conservation, and restoration of declining *Cystoseira* forests.

## 2. Materials and Methods

### 2.1. Specimen Collections and Morphological Identification

Between 2023 and 2024, a total of 250 specimens of *Cystoseira* s.s. were collected from 20 sites across the Atlantic and Mediterranean regions (Table S1; Figure S1). Sampling was designed to capture the broadest possible morphological, ecological, and geographic variation across the range of the target taxa. The selected localities reflect the currently known distribution of these entities, as well as the accessibility of populations during the study period. From each population, approximately 10 thalli located a few metres apart were collected, and for each specimen, approximately 1 g of apical fronds was carefully cleaned of epiphytes and preserved in silica gel for subsequent genetic analyses. For morphological identification, four thalli per population were examined, described and identified using dichotomous keys and morphological descriptions [35,39,40,41,44]. Diagnostic characters were documented photographically, and representative voucher specimens were preserved as exsiccate. Specimens displaying protruding crypts on primary branches were rehydrated, sectioned in a Leica CM1860 cryostat (Leica Biosystem, Nussloch, Germany), and subsequently photographed using a Zeiss Primostar 3 microscope (Carl Zeiss AG, Oberkochen, Germany).

### 2.2. Molecular Analyses

Genomic DNA (gDNA) was successfully extracted from 220 macroalgal specimens using a protocol optimised for polysaccharide-rich macroalgal tissues. Approximately 10 mg of dried tissue was mechanically disrupted using a VWR^®^ Bead Mill homogeniser (VWR International, Radnor, PA, USA) with three stainless-steel beads (⌀ 2 mm). Approximately 2 mg of homogenised tissue powder was then incubated in 20 µL of lysis solution prepared with the PCRBIO Rapid Extract PCR Kit (PCR Biosystems Ltd., London, UK), following the manufacturer’s instructions. Not all collected specimens yielded amplifiable DNA for all markers, resulting in different sample sizes across molecular analyses.

PCR amplification targeted three genetic markers (Table 1): the mitochondrial *cox1* gene, the plastid-encoded *rbcL-rbcS* intergenic spacer (ribulose-1,5-bisphosphate carboxylase/oxygenase, RuBisCO), and the nuclear ribosomal *ITS2* region. The *cox1* fragment was amplified using two primers newly designed in this study, specifically for the genus *Cystoseira* s.l. (COI_CYSTOsl_F and COI_CYSTOsl_R) to minimise the co-amplification of epiphytic contaminants. Despite thorough cleaning of samples prior to DNA extraction, contaminant sequences were initially detected; therefore, genus-specific primers were developed to improve amplification specificity. The fragment including the *rbcL-rbcS* intergenic spacer was retrieved using a primer located on the RuBisCO large subunit (rbcLRH3F; [45]) and one on the RuBisCO small subunit (rbcS139R; [46]). The *ITS2* fragment was amplified using the primer pair 5.8S BF and 25BR2 [47].

The amplification reactions were performed in a final volume of 20 µL containing 10 µL of 2× PCRBIO Ultra Mix (PCR Biosystems Ltd., London, UK), 1 µL of gDNA (diluted 1:100 to 1:1000) and 0.6 µL of each 10 μM forward and reverse primer.

PCR products were checked on 1.5% TAE agarose gels to verify successful DNA amplification and then purified with ExoSAP-IT™ PCR Product Cleanup Reagent (Thermo Fisher Scientific, Waltham, MA, USA) according to the manufacturer’s instructions. The quality and quantity of purified PCR products were assessed with a Nanodrop 2000 spectrophotometer (Thermo Fisher Scientific, Waltham, MA, USA). The final products were Sanger-sequenced by Eurofins Genomics (Ebersberg, Germany).

Out of the 220 extracted specimens, 180 amplified successfully for at least one of the three markers. However, among these 180, 50 were subsequently discarded due to ambiguous chromatograms, double peaks, or very short/low-quality sequences that could not be confidently aligned. Therefore, we obtained 120 usable specimens for at least one marker, yielding a total of 243 new sequences across the three loci.

### 2.3. Phylogenetic and Species Delimitation Analyses

Chromatograms were edited using the software package QIAGEN CLC Genomics Workbench v.25.0.1 (QIAGEN, Hilden, Germany). Sequence identity was verified through BLASTn similarity searches in GenBank [48]. Individual gene alignments were generated with QIAGEN CLC Genomics Workbench, and sequences were manually trimmed to uniform lengths. Outgroups were selected within *Stephanocystis* (Sargassaceae) based on the availability of homologous sequences for each locus. Because the same *Stephanocystis* taxon was not available for all markers, we used the closest available *Stephanocystis* sequences per locus: *S. hakodatensis* (voucher AB043673) for *ITS2*, *S. osmundacea* (voucher AY183897) for *rbcL-rbcS*, and *Stephanocystis* sp. (voucher OK480526) for *cox1*. For *cox1,* the phylogeny of our specimens was inferred in conjunction with selected homologous sequences retrieved from GenBank (complete list provided in Supplementary Information, Table S2). In contrast, for *rbcL-rbcS* and *ITS2*, no sequences of *Cystoseira* s.s. were available in public repositories; therefore, analyses of these markers were based exclusively on sequences generated in this study. To remove redundancy, gene alignments were clustered using CD-HIT [49] with a similarity threshold of 100%. For each alignment, the best-fitting nucleotide substitution model (HKY + G for *cox1*, T92 + G for *rbcL-rbcS*, T92 for *ITS2*) was identified with MEGA11 v.11.0.13 [50]. Phylogenetic trees were inferred using both Bayesian Inference (BI) and Maximum Likelihood (ML) approaches. BI analyses were performed in MrBayes v.3.2.7 [51], with four chains run for 1,000,000 generations, sampling every 1000 generations, yielding 1000 trees. After discarding 25% as burn-in, 750 trees were retained to compute a 50% majority-rule consensus tree with posterior probabilities. ML analyses were run in RAxML-NG v1.2.2 [52]. For each alignment, the best-scoring ML tree was inferred from a random parsimony starting tree under the selected substitution model. Node support was assessed using 100 non-parametric bootstrap replicates, and bootstrap values were mapped onto the final ML tree. In addition to single-locus phylogenies, concatenated alignments were generated to infer multilocus trees. Two concatenated datasets were assembled: one comprising 35 specimens for which all three markers (*ITS2*, *rbcL–rbcS*, and *cox1*) were available, and a second including 79 specimens represented by at least two markers. BI and ML analyses of concatenated datasets were conducted using the same settings described above. Final tree visualisations were created with iTOL: Interactive Tree Of Life [53] to annotate and graphically refine the phylogenies.

To visualise intraspecific variation and the geographic distribution of haplotypes, we reconstructed haplotype networks separately for each single-locus alignment in PopART v1.7 [54], employing the TCS algorithm (Templeton–Crandall–Sing). Alignments were exported in NEXUS format. Gaps (“–”) were treated as informative characters and missing data were coded as “?”. In the resulting networks, each haplotype was represented as a node proportional in size to its frequency, with edges corresponding to the minimum number of mutational steps between haplotypes. Individuals were colour-coded by sampling locality. For the cox1 haplotype network, only sequences generated in this study were included, to ensure full traceability to morphologically validated voucher specimens and to avoid potential biases introduced by misidentified or inconsistently annotated sequences in public repositories.

Species delimitation was assessed using three complementary approaches: ABGD/ASAP (Automatic Barcode Gap Discovery for primary species delimitation, [55]; Assemble Species by Automatic Partitioning [56]), PTP (Poisson Tree Processes; [57]), and GMYC (General Mixed Yule Coalescent; [58]). All methods were applied independently to the *ITS2, rbcL–rbcS,* and *cox1* alignments. ABGD and ASAP analyses were run via the online web server (https://spartexplorer.mnhn.fr/, accessed on 13 March 2025) using both the Jukes–Cantor (JC69) and Kimura two-parameter (K80) substitution models. PTP analyses were based on both ML trees (inferred with RAxML-NG) and Bayesian trees (inferred with MrBayes) using the bPTP web server (http://species.h-its.org/ptp/, accessed on 13 March 2025). For GMYC delimitation, ultrametric gene trees were inferred in BEAST v1.10.5 [59] under all combinations of strict or uncorrelated lognormal relaxed clocks and Yule or coalescent (constant-size) tree priors. Convergence was assessed using Tracer v1.7.1, ensuring effective sample sizes (ESS) greater than 200 for all parameters. Posterior tree distributions were summarised in TreeAnnotator by applying a 10% burn-in and selecting the Maximum Clade Credibility (MCC) tree with mean node heights. The single-threshold GMYC model was then applied to the annotated MCC ultrametric trees to infer species boundaries based on shifts in branching rates using the GMYC web server (https://species.h-its.org/gmyc, accessed on 14 March 2025).

## 3. Results

### 3.1. Alignment Characterisation

A total of 243 new sequences were generated: 99 for *ITS2*, 88 for *cox1,* and 56 for *rbcL-rbcS* (Table S1), from specimens collected at 20 sites across the Mediterranean and Atlantic regions (Table S1, Figure S1). Combined with publicly available data, a total of 325 *Cystoseira* s.s. sequences were analysed, including 82 *cox1* sequences retrieved from GenBank (Table S2). Final single-locus alignments, carefully trimmed to ensure consistent sequence lengths, comprised 1063 bp for rbcL–rbcS, 1046 bp for cox1, and 356 bp for ITS2. Two concatenated multi-locus alignments were also assembled, each 2465 bp in length: the first included 35 specimens for which all three markers were successfully amplified, while the second comprised 79 specimens represented by at least two of the three markers. Among the three markers, *cox1* had the highest proportion of parsimony-informative sites relative to alignment length (65 sites; 6.2%), followed by ITS2 (17 sites; 4.8%) and rbcL–rbcS (22 sites; 2.1%). Due to differential amplification success across markers, the number of sequences for each taxon varied among loci; accordingly, comparisons of intraspecific genetic variation and haplotype diversity across markers were interpreted with this imbalance in mind.

### 3.2. Species Delimitation Methods, Inter-And Intraspecific Diversity and Barcode Gap

Analyses of the three single-locus alignments and their corresponding phylogenetic trees yielded largely congruent results across delimitation methods. Both ABGD/ASAP and PTP consistently recovered two species within *Cystoseira* s.s. (Table 2), corresponding to *C. foeniculacea* and *C. compressa.* The latter included specimens traditionally assigned to *C. humilis* (with the morphology of *C. canariensis*) and *C. pustulata*, hereafter referred to as the *C. compressa* complex. In contrast, GMYC analyses yielded locus-dependent outcomes. A single species was inferred for *ITS2*, two species for *rbcL-rbcS* (*C. foeniculacea* vs. the *C. compressa* complex) and five species for *cox1* (*C. foeniculacea, C. compressa* 1, *C. compressa* 2, *C. humilis,* and *C. pustulata*) (Table 2). This variability highlights the sensitivity of GMYC to marker-specific signal and tree structure, particularly in groups characterised by shallow divergence. This interpretation is further supported by the low per-locus sequence dissimilarity observed among the three entities within the *C. compressa* complex. Mean pairwise dissimilarity ranged from 0.131% to 0.377% in *rbcL-rbcS*, from 0.822% to 0.964% in *cox1*, and from 0.000% to 0.283% in *ITS2*, confirming that divergence within the complex is shallow and marker-dependent. Following Levring’s [36] taxonomic treatment, *C. canariensis* is currently regarded as a synonym of *C. humilis;* however, in our view this conspecificity requires further confirmation (see Discussion). Hereafter, the lineage corresponding to *C. canariensis* (=*C. humilis*?) is referred to as *C.* morphotype *canariensis*.

**Figure 1:**
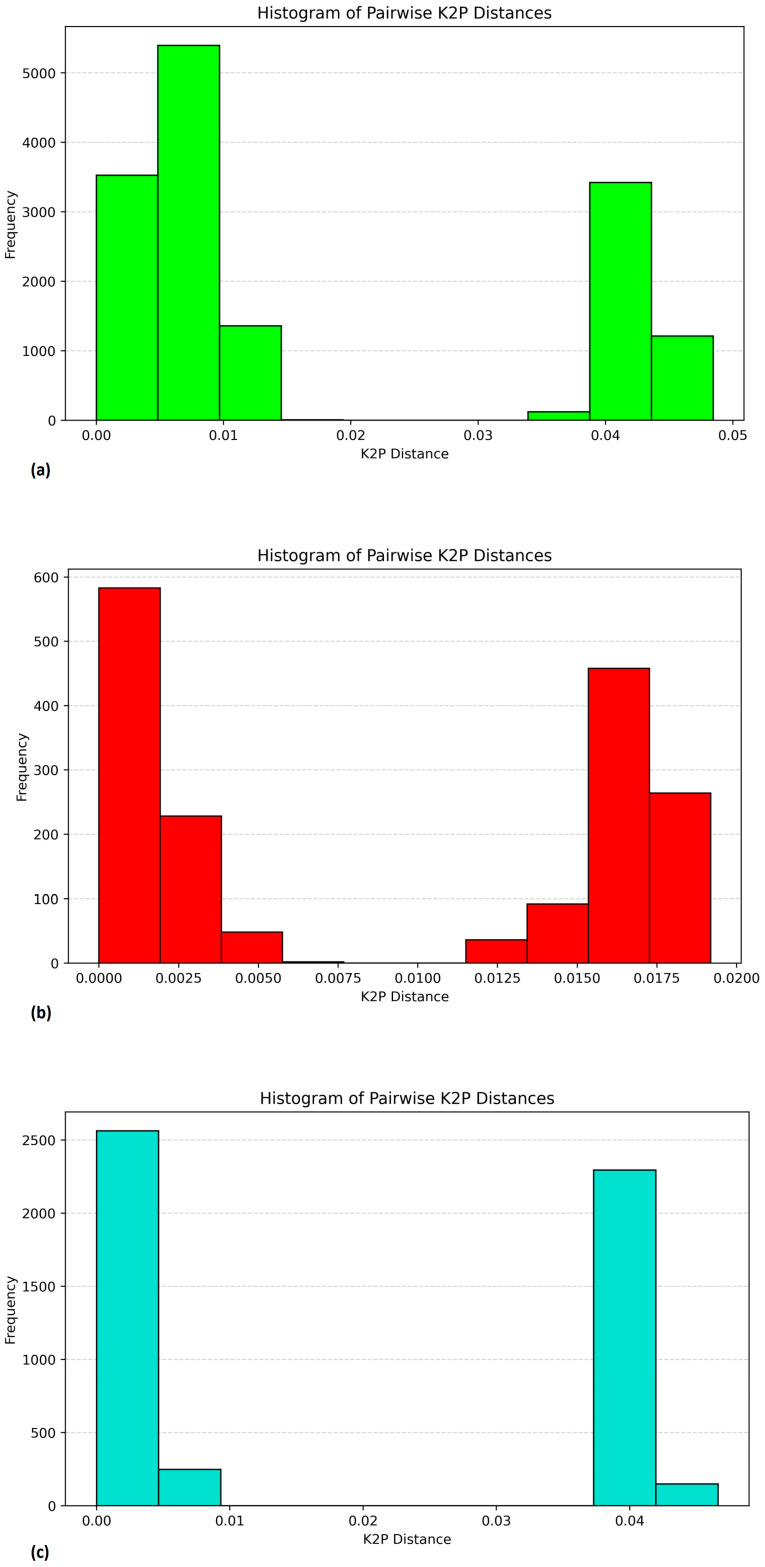
Intra-(left) and interspecific (right) pairwise K2P distances and barcoding gaps in *Cystoseira* s.s. across the three genetic markers: (**a**) cox1 (**b**) rbcL-rbcS (**c**) ITS2.

Considering the most frequently inferred delimitation outcome, namely the recognition of two species (*C. foeniculacea* and the *C. compressa* complex), minimum interspecific genetic distances exceeded maximum intraspecific genetic distances across all three markers, clearly indicating a barcode gap (Figure 1). For *cox1* and *rbcL-rbcS*, minimum interspecific distances were approximately twice the corresponding maximum intraspecific values (0.0359 vs. 0.0156 for *cox1*; 0.0133 vs. 0.0060 for *rbcL-rbcS*). The *ITS2* alignment showed an even greater separation, with inter- and intraspecific distances differing by nearly an order of magnitude (0.0407 vs. 0.0056).

Despite method- and locus-dependent differences in resolution (particularly in GMYC partitions), all three loci consistently supported the same primary split between *C. foeniculacea* and the *C. compressa* complex. This concordance across mitochondrial, plastid, and nuclear markers provides robust evidence for the presence of two main evolutionary lineages within *Cystoseira* s.s., whereas finer subdivisions within the *C. compressa* complex lack consistent support across methods and markers. 

### 3.3. Phylogenetic Trees and Haplotype Networks

The number of haplotypes varied notably among markers, with three detected for *ITS2*, nine for *rbcL-rbcS*, and 29 for *cox1*. Both ML and BI phylogenetic analyses consistently resolved *Cystoseira* s.s. into two well-supported clades, corresponding to *C. foeniculacea* and the *C. compressa* complex.

**Figure 2:**
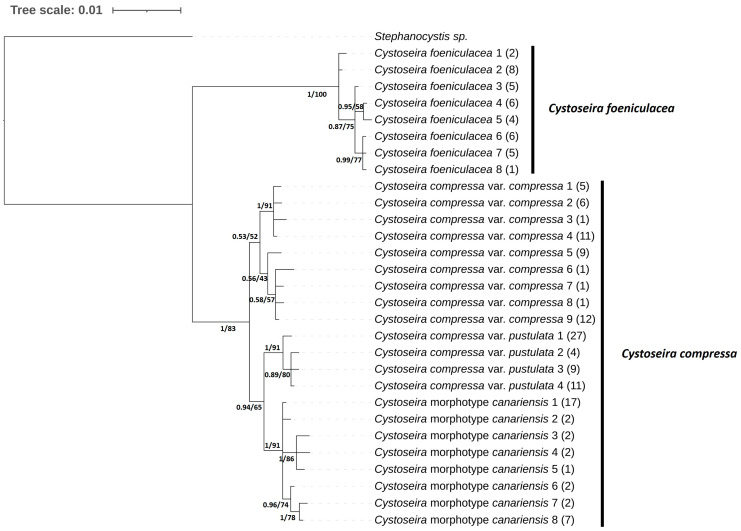
Bayesian Inference (BI) and Maximum Likelihood (ML) consensus tree based on the mitochondrial cox1 marker for *Cystoseira* s.s. Numbers near nodes indicate Bayesian posterior probabilities (left) and ML bootstrap support values (right). Number in parentheses represent the number of identical sequences per haplotype. The complete list of sequences is provided in Supplementary File S1.

The *cox1* phylogenetic trees (Figure 2) recovered eight haplotypes for *C. foeniculacea* and 21 for the *C. compressa* complex, encompassing specimens from across the Atlantic and Mediterranean regions (Spain, Portugal, Italy, Croatia, France, Morocco, Israel, Greece, Malta).

Within the *C. compressa* complex, some clades were recovered only as short branches with low support, suggesting subtle but weakly supported genetic differentiation. These patterns indicate limited phylogenetic resolution among closely related haplotypes and shallow genetic differentiation, consistent with recent divergence and/or incomplete lineage sorting.

**Figure 3:**
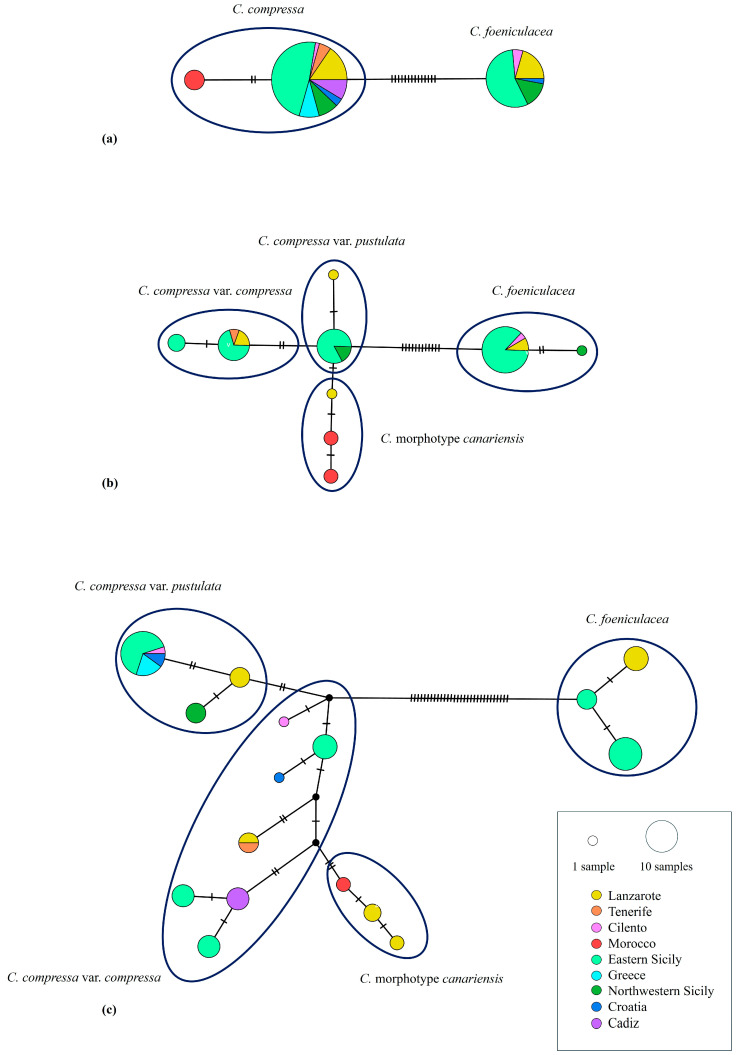
Haplotype networks of *Cystoseira* sensu stricto based on single-locus datasets. (**a**) ITS2 haplotype network (**b**) rbcL–rbcS haplotype network (**c**) cox1 haplotype network, based exclusively on sequences generated in this study. In all networks, each circle represents a unique haplotype, with circle size proportional to haplotype frequency. Small black dots indicate unsampled or inferred intermediate haplotypes. Ticks on the lines connecting haplotypes represent single mutational steps. Haplotypes are colour-coded according to sampling locality, illustrating the geographic distribution of genetic variation within and among lineages. Across all loci, haplotypes cluster into two main groups corresponding to *Cystoseira foeniculacea* and the *C. compressa* complex, while internal structure within the *C. compressa* complex is characterised by shallow divergence and limited haplotype separation.

**Figure 4:**
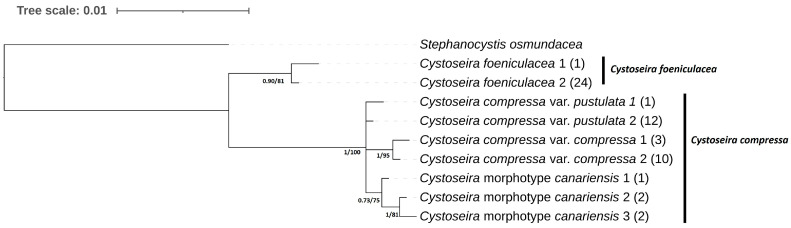
Bayesian Inference (BI) and Maximum Likelihood (ML) tree based on the plastidial rbcL-rbcS intergenic spacer for *Cystoseira* s.s. Numbers near the nodes indicate Bayesian posterior probabilities (left) and ML bootstrap support values (right). Number in parentheses represents the number of identical sequences per haplotype. The complete list of sequences is provided in Supplementary File S2.

**Figure 5:**
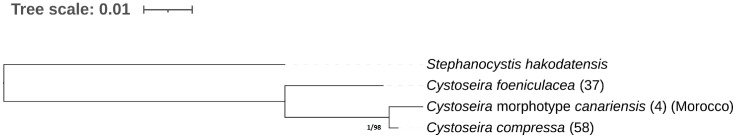
Bayesian Inference (BI) and Maximum Likelihood (ML) consensus tree based on the nuclear ribosomal ITS2 region for *Cystoseira* s.s. Numbers near the nodes are Bayesian posterior probabilities (left) and ML bootstrap support values (right). Number in parentheses represents the number of identical sequences per haplotype. The complete list of sequences is provided in Supplementary File S3.

The *cox1* haplotype network (Figure 3c), based exclusively on sequences generated in this study, further illustrates the close genetic relationships within the complex, with only a small number of mutational steps separating haplotypes and no clear discontinuities indicating discrete haplogroups. The *rbcL–rbcS* phylogenies and corresponding haplotype network (Figure 4 and Figure 3b) identified two haplotypes for *C. foeniculacea* and seven for the *C. compressa* complex, within the latter, the three morpho-ecological entities were connected through an unresolved node (Figure 4). In contrast, *ITS2* phylogenies recovered only a single haplotype for *C. foeniculacea* and two for the *C. compressa* complex, one of which comprised exclusively *C.* morphotype *canariensis* specimens from the Atlantic coast of Morocco (Figure 5 and Figure 3a).

**Figure 6:**
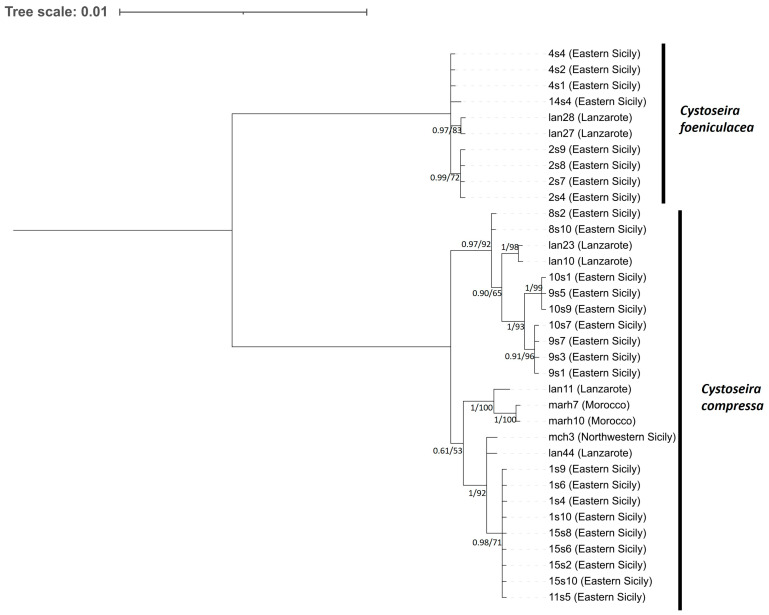
Multilocus Bayesian Inference (BI) and Maximum Likelihood (ML) consensus tree based on the concatenated dataset (ITS2 + rbcL–rbcS + cox1) for *Cystoseira* s.s. GenBank accession numbers are listed in Table S1. Numbers near nodes indicate Bayesian posterior probabilities (left) and ML bootstrap support values (right).

ML and BI analyses of the concatenated multilocus alignment based on the larger dataset of 79 specimens (each represented by at least two markers) failed to resolve internal relationships within the *C. compressa* complex, which appeared as an unresolved node in the Bayesian phylogeny (Figure S2). We attribute this result to the amount of missing data inherent in this larger dataset. By contrast, restricting the analysis to the 35 specimens with complete multilocus data (Figure 6, Table S1) produced a robust topology that closely mirrored the *cox1* phylogeny (Figure 2).

Across all analyses, two strongly supported clades were consistently recovered, corresponding to *C. foeniculacea* and the *C. compressa* complex; within an integrative framework incorporating molecular, morphological, and ecological evidence (see Table S3), the latter is best interpreted as comprising the autonym plus two additional intraspecific lineages treated here at varietal rank (var. *pustulata* and *C.* morphotype *canariensis*).

### 3.4. Morphological Analyses and Ecological Observations

#### 3.4.1. *Cystoseira foeniculacea* (Linnaeus) Greville

Specimens resolved by molecular data as *C. foeniculacea* corresponded morphologically to either the autonym C. *foeniculacea* f. *foeniculacea, C. foeniculacea* f. *latiramosa* (Ercegović) Gómez Garreta, Barceló, Ribera & Rull Lluch or *C. foeniculacea* f. *tenuiramosa* (Ercegović) Gómez Garreta, Barceló, Ribera & Rull Lluch. Despite their clearly distinct morphologies, no genetic differentiation was detected among specimens attributable to the three morphological forms; accordingly, they are collectively referred to as *C. foeniculacea* in the phylogenetic trees and haplotype networks presented herein.

**Figure 7:**
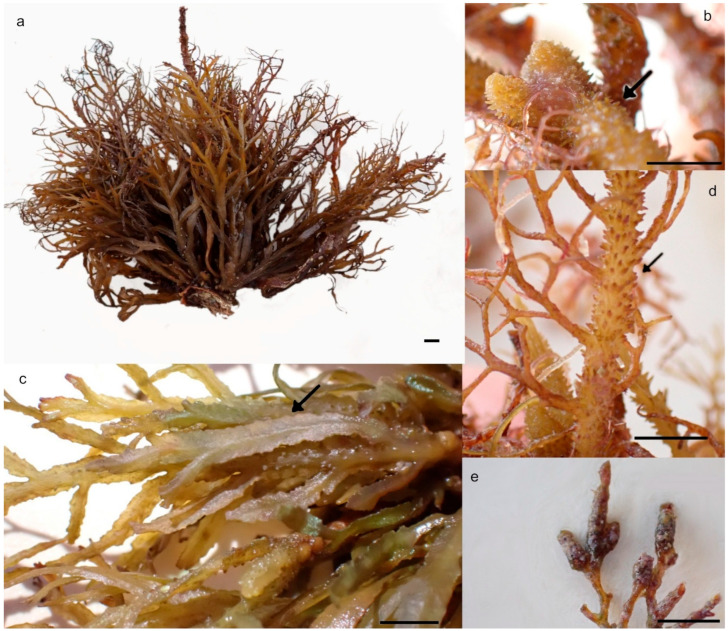
*Cystoseira foeniculacea*: (**a**) habit; (**b**) slightly protruding apex with small thorns; (**c**) compressed branches with crenate to denticulate margins and conspicuous midrib; (**d**) primary branches with numerous thorns; (**e**) receptacles. Scale bar = 1cm.

Thalli of *C. foeniculacea* f. *foeniculacea* have a conical to discoid holdfast, from which cylindrical cauloids arise (Figure 7a). Apices are slightly protruding and bear small thorns (Figure 7b). Some primary branches are compressed, with crenate to denticulate margins and a conspicuous median rib (Figure 7c). The most distinctive feature of this form, the roughened surface of the primary axes due to numerous thorns, was consistently observed (Figure 7d). Receptacles are elongate, cylindrical, simple or occasionally forked, occurring either solitary or in compact clusters (Figure 7e).

Thalli assignable to *C. foeniculacea* f. *latiramosa* exhibit compressed primary and secondary branches with conspicuously toothed margins and a distinct median rib, whereas *C. foeniculacea* f. *tenuiramosa* is characterised by cylindrical cauloids and primary branches, with higher-order axes filiform and delicate.

No specimens referable to *Cystoseira foeniculacea* f. *schiffneri* (Hamel) Gómez Garreta, Barceló, Ribera & Rull Lluch, reinstated at species rank as *C. schiffneri* Hamel by Bouafif et al. [38] (p. 144), were detected in our samples. Both its taxonomic reinstatement and its classical record from the Stagnone di Marsala, Sicily (reported in Giaccone et al. [60] as *C. ercegovicii* Giaccone nom. inval.) are currently based exclusively on morphological evidence in the absence of molecular data. Targeted resampling and sequencing at type localities will be required to resolve its taxonomic status and geographic distribution.

#### 3.4.2. *Cystoseira Compressa* Complex

DNA-based assignments placed specimens within the *C. compressa* complex whose morphologies corresponded to the traditional diagnoses of *C. compressa*, *C. pustulata*, and *C. canariensis* (traditionally treated as a synonym of *C. humilis*, but see above), as well as several intermediate morphotypes. In some cases, diagnostic traits traditionally used to distinguish *C. canariensis* (=*C. humilis*?) from *C. pustulata*, or *C. compressa* from *C. pustulata*, were weakly expressed, overlapping or combined in intermediate ways, making unambiguous assignment based on morphology alone difficult. The morphological accounts provided below (see also Table S3) should therefore be regarded as representing the *typical* expressions of each variety rather than discrete, non-overlapping character sets. Although the three entities show recurrent and recognisable morpho-ecological patterns, considerable phenotypic variability occurs both within and among populations, and intermediate morphologies frequently encountered. This partial morphological overlap, together with the shallow genetic divergence detected among entities, supports their interpretation as varieties rather than distinct species.

#### 3.4.3. *C. compressa* Var. *compressa*

Thalli assigned to the autonym exhibited the diagnostic characters of the basionym *Fucus compressus* Esper, later transferred to the genus *Cystoseira* by Gerloff and Nizamuddin [61] (p. 342) as *C. compressa*.

**Figure 8:**
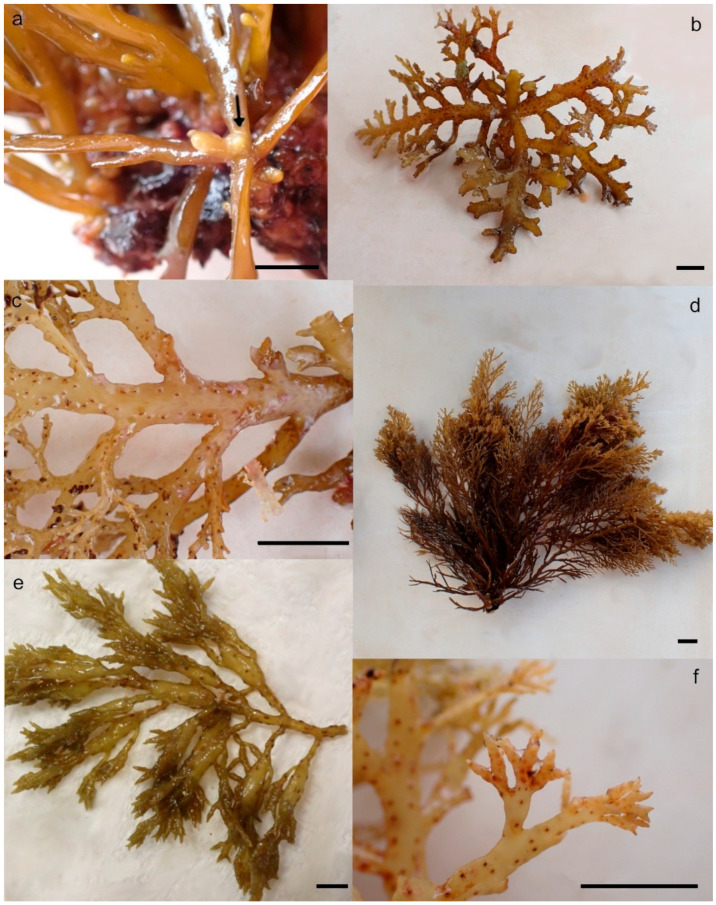
*Cystoseira compressa* var. *compressa* (**a**) smooth and prominent apex; (**b**) winter habit; (**c**) branches with prominent cryptostomata; (**d**) spring-summer habit; (**e**) aerocysts; (**f**) terminal receptacles. Scale bars = 0.5 cm (**a**,**c**,**e**,**f**) and 1 cm (**d**).

Thalli are caespitose, attached by a relatively small, compact, discoid holdfast; cauloids are short (1–3 cm), cylindrical or slightly flattened, with smooth and prominent apices (Figure 8a), often ramified near the base. Fronds are yellowish- to dark-brown, non-iridescent. Frond morphology follows a marked seasonal trajectory [35,62], consistently observed in both Mediterranean and Atlantic specimens. During winter, thalli are reduced, forming short, flattened, distichously branched primary axes arranged in a rosette-like habit (Figure 8b) with smooth margins and rounded apices; a distinct midrib is visible in both primary and secondary axes, and cryptostomata are arranged in two parallel rows from base to apex (Figure 8c). In spring–summer, thalli become erect, well-developed and highly ramified, particularly in the apical region (Figure 8d). Primary axes, flattened at the base and cylindrical distally, elongate to 20–40 cm, reaching up to 1 m in highly sheltered sites. Secondary and higher-order branches are slender (1.5–2 mm), cylindrical, simple or sparsely branched, frequently bifid, and arranged alternately and distichously. Cryptostomata are abundant, regularly distributed around the circumference, not, or only slightly, protruding, and already visible on primary axes. Aerocysts are usually present (Figure 8e), numerous, and variable in size, ranging from small (3–6 × 1–2 mm) to large (8–10 mm), mostly subterminal and isolated or arranged in series of two to three. Receptacles are terminal, compact, fusiform to lanceolate-fusiform (1–10 × 0.5–1 mm), simple or bi- to trifurcate (Figure 8f), and occasionally briefly pedicellate on aerocysts.

Thalli typically inhabit the upper infralittoral zone (0–5 m), occurring in both exposed and sheltered areas (including bay-heads), and are also found in intertidal rock pools. Exposure to wave action strongly influences thallus size, with plants remaining small in wave-exposed sites but growing large (50–60 cm) in sheltered localities.

#### 3.4.4. *C. compressa* Var. *pustulata* Comb. Nov.

**Figure 9:**
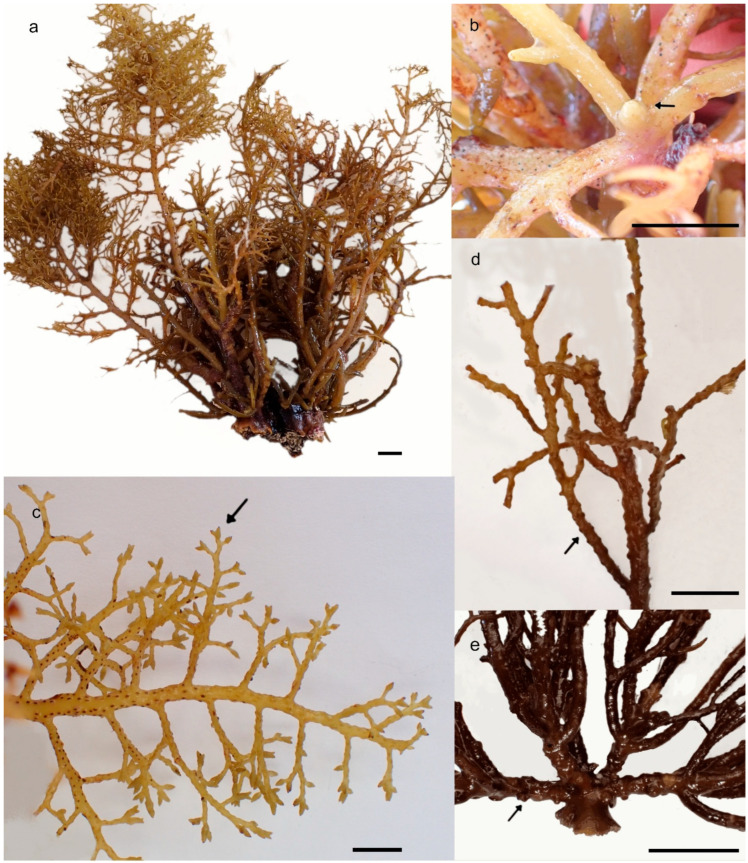
*Cystoseira compressa* var. *pustulata* (**a**) habit; (**b**) smooth and prominent apex; (**c**) terminal receptacles; (**d**) branches with prominent cryptostomata; (**e**) base of cauloids with protuberances from the stumps of deciduous branches. Scale bars = 1 cm (**a**,**c**,**e**) and 0.5 cm (**b**,**d**).

Thalli morphologically examined and molecularly assigned to *C. compressa* var. *pustulata* are characterised by a more slender and delicate habit than *C. compressa* var. *compressa*, with cylindrical branching, and closely match the description of *C. abrotanifolia* (Linnaeus) C. Agardh subsp. *pustulata* Ercegović [39]. Plants form erect fronds, usually 10–30 cm high, yellowish to light brown, and never iridescent. A small, compact, discoid holdfast gives rise to short cauloids (1–3 cm) bearing smooth, slightly protruding apices (Figure 9a,b). In some specimens, basal protuberances corresponding to scars left by shed deciduous branches are evident (Figure 9e). Primary branches are mostly cylindrical or slightly compressed at the base (2–2.5 mm wide) and often begin branching in their lower half. Secondary and higher-order branches are slender (1.5–2 mm), cylindrical, simple or sparsely branched, and may occasionally be arranged distichously. Cryptostomata are abundant (Figure 9d), conspicuous, and evenly distributed around the circumference of all axes, already evident on primaries; they are never pedicellate. Aerocysts are usually absent, or, when present, very small (2–3 mm). Receptacles are terminal, compact, fusiform (1–2 × 0.5 mm), simple or rarely bifurcate (Figure 9c). In contrast to var. *compressa*, no pronounced seasonal phenological variation was observed. During autumn-winter, fronds remain cylindrical, short and weakly developed, never forming the *rosette-like* habit, whereas vigorous growth occurs in spring-summer, in agreement with the original observations of Ercegović [39]. This variety inhabits shallow infralittoral rocky substrates (0.5–5 m) and tide pools; in the Mediterranean, it generally occurs deeper than var. *compressa*, sometimes reaching 20–30 m.

#### 3.4.5. *Cystoseira* Morphotype *canariensis*

All specimens molecularly assigned to *C.* morphotype *canariensis* correspond morphologically to *C. canariensis* as described by Sauvageau [40] (p. 468).

**Figure 10:**
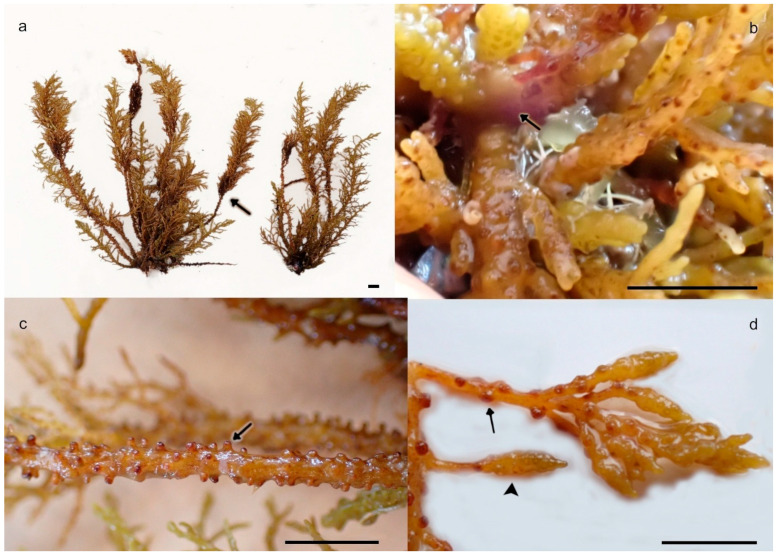
*Cystoseira* morphotype *canariensis*: (**a**) habit with secondary branches arising from the upper two-thirds of the primary branches; (**b**) slightly protruding apex; (**c**) primary branches with conspicuous pedicellate, truncated cryptostomata; (**d**) receptacles (arrowhead) and crypts (arrow). Scale bars = 1 cm (**a**,**d**) and 0.5 cm (**b**,**c**).

**Figure 11:**
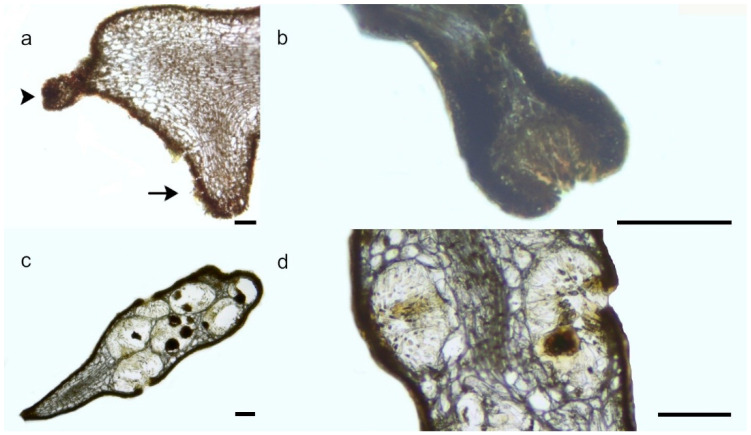
*Cystoseira* morphotype *canariensis* sections: (**a**) cryptostomata (arrowhead) and branch (arrow); (**b**) cryptostomata; (**c**) receptacle; (**d**) detail of receptacle. Scale bar = 0.14 mm.

Thalli form turfs, are never iridescent and completely lack aerocysts (Figure 10a). They are attached by a compact discoid holdfast, from which several short, stout, erect cauloids (1–3 cm long) arise with smooth, slightly protruding apices (Figure 10b). Fallen branches leave prominent circular scars that do not give rise to adventitious shoots. Two to five erect primary branches (10–25 cm long) develop, strictly cylindrical and never flattened, (even at juvenile stages), progressively attenuating towards the apices; basal portions become rapidly denuded during growth. Primary branches bear numerous cylindrical crypt-bearing pedicels (0.4–0.6 mm long), a diagnostic feature of this entity (Figure 10c and Figure 11a,b). These pedicels, abruptly truncated and terminating in a single piliferous crypt, represent the most distinctive morphological trait separating this entity from the other varieties within the complex. Secondary branches bear abundant crypts with markedly protruding ostioles, imparting a distinctly nodose surface (Figure 10d). First-order laterals are slender, short, and simple, whereas second-order branches reach 2–4 cm, are frequently branched once or twice, and gradually decrease in size distally. Tertiary laterals are delicate, often curved, and terminate in short pedicels. Laterals are inserted irregularly, although tertiary axes tend to be arranged in two opposite rows. Fertility is restricted to the terminal portions of the primary branches; secondary branchlets in these distal zones bear receptacles of variable size and form, simple or sparingly branched, slightly tuberculate to fusiform (Figure 10d and Figure 11c,d).

Ecologically, this variety is confined to the Atlantic, where it inhabits shallow intertidal rock pools in the Canary Islands. In deeper and more permanently submerged pools, thalli exhibit a dwarf growth form, with cauloids approximately 1 cm long and primary branches 2–5 cm in length, bearing scattered or distichous secondary laterals, often simple, with abundant protruding crypts and terminal receptacles. Specimens collected in Morocco are more robustly developed and occur in shallow rock pools of the upper intertidal zone.

## 4. Discussion

### 4.1. Evidence for Two Species-Level Lineages and Intraspecific Diversification in Cystoseira s.s.

Our multilocus phylogenetic analyses support the recognition of two species-level lineages within *Cystoseira* s.s., corresponding to *C. foeniculacea* and *C. compressa*. Within *C. compressa*, both C. *pustulata* and *C.* morphotype *canariensis* (i.e., specimens with the morphology traditionally attributed to *C. canariensis*) are best interpreted as intraspecific lineages rather than independent species. Accordingly, we propose their treatment at varietal rank, while formally establishing a new combination only for *C. pustulata*, pending clarification of the conspecificity between *C. canariensis* and *C. humilis*. This pattern is consistent with a scenario of recent or ongoing diversification, in which morpho-ecological differentiation has progressed further than genomic lineage sorting at the analysed molecular markers. This taxonomic interpretation is primarily based on the pattern of genetic discontinuity observed across the three molecular markers. The barcode gap identified in our dataset (Figure 1) consistently separates *C. foeniculacea* from the *C. compressa* complex, with interspecific divergence markedly exceeding the range of intraspecific variation. By contrast, pairwise divergences among the three morpho-ecological entities within the *C. compressa* complex remain low across all markers falling far below the interspecific threshold that distinguishes the two main lineages (see Results 3.2).

In this study, the use of multiple species-delimitation methods, which could be sensitive to sampling density and phylogenetic reconstruction uncertainty, was intended not as stand-alone criteria for taxonomic decisions, but as a comparative tool to test the robustness of inferred boundaries across markers. Their inclusion was also motivated by the fact that recent taxonomic changes within *Cystoseira* s.s., including the elevation of *C. pustulata* to species rank [27], relied in part on similar analytical frameworks. In this context, both PTP and ABGD/ASAP consistently recovered only two species across all three markers, whereas GMYC yielded highly locus-dependent outcomes, ranging from one to five putative species. The least conservative GMYC partition would imply splitting *Cystoseira* s.s. into five entities, exceeding the four taxa previously recognised; conversely, the most conservative outcome collapsed all *Cystoseira* s.s. into a single species. Such instability reflects a well-documented limitation of GMYC, which is prone to oversplitting in datasets characterised by shallow divergence, incomplete lineage sorting, or large alignments [63,64]. Accordingly, GMYC results are best interpreted here as an exploratory signal of divergence heterogeneity, highlighting putative cryptic lineages, rather than as evidence for formal species recognition. Talavera et al. [64] reported substantial sensitivity and failure rates under several conditions, concluding that GMYC partitions should not be equated with species *sensu stricto*, but instead regarded as “candidate” species requiring corroboration. By contrast, the strong congruence between ABGD/ASAP and PTP results, combined with the recovery of a clear and consistent barcode gap between inter- and intraspecific distances across all three markers, provides robust support for recognising only two species-level lineages within *Cystoseira* s.s. Our taxonomic interpretation does not rest solely on these delimitation methods, but on the combined evaluation of diagnosability, phylogenetic relationship, and the absence of evidence for independently evolving species-level lineages within the *C. compressa* complex. In all cases, the barcode gap consistently observed in *cox1, rbcL–rbcS*, and *ITS2* indicates that genetic divergence between the two main lineages (*C. foeniculacea* vs. the *C. compressa* complex) consistently exceeds variation within each lineage.

Our results contrast with those of Neiva et al. [27], who relied exclusively on *cox1* and elevated *C. compressa* subsp. *pustulata* to species rank despite the absence of a clear barcode gap and inconsistent outcomes across the delimitation methods applied. Instead, our findings are more consistent with de Sousa et al. [25], who observed that the autonym of *C*. *compressa* branched off from *C*. *compressa* subsp. *pustulata* and *C*. *humilis* without significant support values, acknowledging the close phylogenetic affinity of the latter two and proposing their synonymy.

Additional instability is evident in the treatment of *C. aurantia* Kützing by Orellana et al. [1], later re-identified as *C. pustulata* by Neiva et al. [27]. Our analyses instead place these sequences closer to *C. compressa* var. *compressa*, further illustrating how shallow divergence and morphological plasticity can lead to inconsistent taxonomic interpretations when single markers are used in isolation.

### 4.2. Morphological Plasticity and Consistent Ecological Differentiation Within the C. compressa Complex

Genetic distances among the three entities traditionally recognised as *C. compressa*, *C. pustulata*, and *C.* morphotype *canariensis* are consistently low and often marker-dependent (Figure 2, Figure 3, Figure 4 and Figure 5). This pattern mirrors the morphological evidence, which reveals pronounced plasticity in diagnostic traits, such as branch compression, presence and distribution of aerocysts, and cryptostomata structure, both in our material and in previous studies [16,35,38,39,40]. Expression of these traits frequently overlaps among populations in our material, and previous taxonomic studies have suggested that their development may vary in relation to environmental conditions such as light availability, hydrodynamic exposure, and tidal regime [16,35,38,39,40]. For instance, aerocysts or cryptostomata may be conspicuous in sheltered subtidal habitats but strongly reduced or absent in highly exposed intertidal sites, blurring the distinction between *C. compressa* var. *compressa* and *C. compressa* var. *pustulata.* Such environmentally mediated phenotypic variability has historically fuelled inconsistent taxonomic interpretations and contributed to species inflation within the complex. From a cladistic standpoint, none of these traits constitutes a fixed apomorphy: their continuous, overlapping variation does not provide the discrete character-state discontinuities expected for independently evolving species-level lineages.

Remarkably similar patterns have been documented in other Fucales, such as in *Sargassum* from the Mexican Atlantic, where molecular analyses collapsed ten morphologically distinct species into a single polytomy with very low genetic divergence [65].

The taxonomic status of *C. humilis* and *C. canariensis* is particularly emblematic of this problem. *C. canariensis* was originally described by Sauvageau [40] from the Canary Islands as forming dense turfs in shallow rock pools, with short erect cauloids, strictly cylindrical primary branches, and pedicellate cryptostomata. Although Sauvageau regarded it as distinct, Levring [36] (p.81; see also Price et al., [37]) later treated it as conspecific with *C. humilis.* Nevertheless, none of the morpho-anatomical accounts of *C. humilis* (e.g., [35,41,42,44]) explicitly report the distinctive diagnostic suite of characters observed in our material, which closely matches Sauvageau’s original description of *C. canariensis*.

Despite this variability and partial overlap in individual traits, our integrative framework reveals three morpho-ecological patterns that are consistently recognisable across broad geographic and ecological gradients. *C. compressa* var. *compressa* develops larger fronds with basal flattening and aerocysts, dominating intertidal and upper subtidal zones. *C. compressa* var. *pustulata* occupies shallow subtidal habitats down to 10–20 m and is characterised by slender, cylindrical fronds with protruding cryptostomata and limited seasonal phenological variation. *C.* morphotype *canariensis* forms dense turfs restricted to the Atlantic, characterised by pedicellate cryptostomata and confinement to tide pools and sheltered environments.

Crucially, although these entities exhibit morphological and ecological differentiation, the boundaries between them are not sharply discrete, as some specimens display intermediate character states and cannot be assigned unambiguously on the basis of morphology alone. In this context, morphology supports differentiation within the complex, but does not reveal a level of discontinuity comparable to that observed between the two main species-level lineages (i.e., *C. foeniculacea* vs. the *C. compressa* complex). This combination of evidence argues against their recognition as independent species under a phylogenetic species concept, while fully justifying their treatment as varieties. Recognition at variety rank therefore acknowledges ecologically meaningful diversity while avoiding unsupported species inflation.

In this sense, *C. compressa* var. *compressa*, *C. compressa* var. *pustulata*, and *C.* morphotype *canariensis* represent stable, geographically and ecologically coherent taxonomic units that reconcile phenotypic distinctiveness with a conservative and evolutionarily sound interpretation of species boundaries.

### 4.3. Performance of Molecular Markers and Implications for Future Taxonomic Research

Among the three markers used, *cox1* emerged as the most variable locus, in agreement with previous studies on *Cystoseira* s.l. and related fucoid algae [1,17,25,27,32]. Although additional loci such as *23S rDNA*, *23S–tRNA^Val*, *psbA*, *nad1*, and *psaA* have been employed in earlier phylogenetic studies, these markers have generally shown limited resolution at the species-level and were therefore not included in the present analyses. Instead, we combined *cox1* with two non-coding regions, the plastid rbcL–rbcS intergenic spacer and the nuclear *ITS2* region, both of which have been successfully applied for species delimitation and intraspecific assessments in Phaeophyceae [30,33,34,47]. The rationale for this multilocus design was to integrate markers evolving at different rates and under different inheritance models, thereby maximising the likelihood of detecting both deep and shallow evolutionary signals. The lower variability observed in *rbcL–rbcS* and *ITS2* does not necessarily imply poor taxonomic resolution, as a few base pair differences may reflect strong sequence conservation rather than insufficient discriminatory power.

The congruent recovery of two main evolutionary lineages across mitochondrial, plastid, and nuclear markers demonstrates that a multilocus framework provides a more reliable basis for taxonomic decisions than single-locus approaches, particularly in groups characterised by shallow divergence, morphological plasticity, and potential hybridisation.

Nevertheless, amplification success rates varied considerably among markers, reflecting both the inherent difficulties of working with brown algal DNA and differences in primer specificity. The particularly low yield for *rbcL-rbcS* is largely attributable to the use of primers originally designed for other algae (i.e., Rhodophyta for the forward primer and Ectocarpales, Phaeophyceae for the reverse primer). Their suboptimal specificity may have facilitated the preferential amplification of co-extracted epiphytic DNA over that of the target. These technical challenges were compounded by the intrinsic properties of brown macroalgae, which are rich in polysaccharides and polyphenols that interfere with DNA extraction and PCR amplification, particularly when field preservation conditions are suboptimal. Despite careful cleaning of all specimens prior to processing, microscopic or cryptic epiphytes can be easily overlooked, often resulting in mixed chromatograms and unusable sequences.

Looking ahead, taxonomic work on *Cystoseira* s.l. should prioritise the development of additional nuclear markers and the design of genus-specific primers, as well as optimising extraction protocols to enhance template quality, thereby improving amplification success and phylogenetic resolution. While mitochondrial *cox1* remains highly informative, its resolution alone is insufficient to disentangle recent or ongoing divergence processes. Expanding the suite of independent nuclear loci therefore represents a critical first step towards stabilising species boundaries. The multilocus approach adopted here has provided a valuable baseline framework; nevertheless, like any marker-limited approach, it does not permit the detection of heterozygosity, recombination, or fine-scale population structure which are aspects that would require higher genomic coverage. A transition toward genomic-scale data, such as RAD-seq, whole-genome resequencing, or target enrichment approaches, will be particularly valuable for quantifying admixture in zones of co-occurrence, testing hypotheses of ecological differentiation, and detecting ongoing gene flow within the *C. compressa* complex.

Another pressing issue concerns the misidentification of specimens in earlier studies, which has resulted in incorrectly named sequences in public repositories such as GenBank and, consequently, unstable phylogenetic reconstructions. Systematic re-evaluation of existing databases, anchored to morphologically validated reference material, is urgently needed. Indeed, in recent years, similar initiatives have also been proposed for other macroalgal groups [66]. Equally essential is the molecular analysis of type material using standardised and reproducible protocols. The absence of genetic data from nomenclatural types continues to hinder the resolution of long-standing taxonomic ambiguities in *Cystoseira* s.l. and integrating DNA data from historical collections with rigorous morphological reassessment will be indispensable to firmly anchor molecular clades to valid names.

Finally, beyond *Cystoseira* s.s., comprehensive reassessment of other segregated genera (e.g., *Ericaria, Gongolaria*) within an integrative multilocus framework is required to establish a coherent taxonomy for the entire clade. Coordinated efforts, combining new markers, curated sequence databases, analyses of type material, and fine-scale population studies will be essential to stabilise the taxonomy of *Cystoseira* s.l. and to define genotype-phenotype-environment relationships, thereby providing a robust foundation for biodiversity monitoring, ecological research, and conservation of these foundational marine forests.

### 4.4. Nomenclatural Novelties

The congruence of molecular, morphological, and ecological evidence supports the recognition of only two species-level lineages within *Cystoseira* s.s., corresponding to *C. foeniculacea* and *C. compressa*. Within *C. compressa*, however, consistent morpho-ecological differentiation warrants formal taxonomic recognition below the species rank, while remaining fully compatible with the shallow and marker-dependent genetic divergence observed.

Accordingly, two varieties in addition to the autonym are recognised within *C. compressa*, only one of which is formally proposed here, pending clarification of the taxonomic status and typification of *C. canariensis* relative to *C. humilis*. This conservative approach ensures nomenclatural stability while acknowledging ecologically meaningful diversity.

*Cystoseira compressa* (Esper) Gerloff et Nizamuddin var. *pustulata* (Ercegović) D’Ambros Burchio & Falace comb. et stat. nov.

Basionym: *Cystoseira abrotanifolia* subsp. *pustulata* Ercegović 1952: 113. Fauna et Flora Adriatica. Sur les *Cystoseira* adriatiques. Leur morphologie, écologie et évolution. Vol. 2: 1–212. Institut d’ Océanographie et de Pêche, Split.

Homotypic synonym: *Cystoseira compressa* subsp. *pustulata* (Ercegović) Verlaque.

Note: Based on combined morphological, reproductive, ecological, and molecular evidence, recognition at varietal rank is considered more appropriate than subspecies or species rank, as it captures consistent phenotypic differentiation without implying unsupported evolutionary independence.

It should also be noted that Verlaque [67] (p. 191) published the name *C. compressa* var. *pustulata* Ercegović (sic!), without explicitly designating it as a new combination; consequently, that name, which should be credited to Ercegović ex Verlaque, is invalidly published.

## Figures and Tables

**Table 1:** Primers used to amplify gDNA, alongside their sequences, references and PCR conditions.

Primer Name	Primer Sequence 5′→3′	Amplicon Length (bp)	Source	Thermal Profiles
COI_CYSTOsl_F/COI_CYSTOsl_R	F: 5′-GGTACMGCGATGTCYGTTCT-3′ R: 5′-TGNCCTAAAATYTCHGGATA-3′	1140	This study	95 °C 1′; 40 cycles: 95 °C 15″, 55 °C 30″, 72 °C 1′; 72 °C 5′
rbcLRH3F/rbcS139R	F: 5′-TTAAYTCTCARCCDTTYATGCG-3′ R: 5′-AGACCCCATAATTCCCAATA-3′	1155	[45,46]	95 °C 1′; 40 cycles: 95 °C 15″, 52 °C 20″, 72 °C 1′; 72 °C 5′
5.8S BF/25 BR2	F: 5′-GATGAAGAACGCAGCGAAATGCGAT-3′ R: 5′-TCCTCCGCTTAGTTATATGCTTAA-3′	467	[47]	95 °C 1′; 33 cycles: 95 °C 15″, 62 °C 20″, 72 °C 30″; 72 °C 5′

**Table 2:** Results of species delimitation methods. The table reports the number of putative species inferred from each marker using ABGD/ASAP, PTP, and GMYC.

	ABGD/ASAP		PTP		GMYC			
	JC69	K80	ML	Bayes	SC	SY	RC	RY
***ITS2***	2	2	2	2	1	1	1	1
***rbcL-rbcS***	2	2	2	2	2	2	2	2
***cox1***	2	2	2	2	5	5	5	5

JC69: Jukes-Cantor distance; K80: Kimura two-parameter distance; PTP ML: Maximum Likelihood partition; PTP Bayes: Bayesian partition with highest posterior support; GMYC S/R: strict or relaxed molecular clock; C/Y: coalescent (constant size) or Yule tree prior.

## Data Availability

The data presented in this study are available in the article and Supplementary Materials. The genetic sequences generated in this study have been deposited in GenBank under the accession numbers reported in the Supplementary Materials and will become publicly available upon publication.

## Supplementary Materials

The following supporting information can be downloaded at: https://www.mdpi.com/article/10.3390/plants15142237/s1. Figure S1: Sampling sites across the Atlantic Ocean (panel A) and Mediterranean Sea (panel B). Figure S2: Multilocus Bayesian Inference (BI) and Maximum Likelihood (ML) consensus tree based on the concatenated dataset (at lea*ITS2* + *rbcL–rbcS* + *cox1*) with missing data for *Cystoseira* s.s. Numbers near nodes indicate Bayesian posterior probabilities (left) and ML bootstrap support values (right). Table S1: List of samples used for phylogenetic and morphologic analyses; Table S2: List of additional sequences used for the cox1 phylogenetic tree and species delimitation methods; Table S3: Morphological characters of the three varieties of Cystoseira compressa. File S1: sequence clustered in the cox1 phylogenetic tree; File S2: sequence clustered in the rbcL-rbcS phylogenetic tree; File S3: sequence clustered in the ITS2 phylogenetic tree.

## Abbreviations

*cox1*: cytochrome oxidase 1; *ITS2*, internal transcribed spacer 2; RuBisCO, ribulose-1,5-bisphosphate carboxylase/oxygenase; *rbcL-rbcS*, RuBisCO large-small subunits; *psbA*, photosystem II protein D1; *mt23S*, mitochondrial 23S; *mt23S–tRNA^Val*, mitochondrial 23S–tRNA^Val spacer; PCR, Polymerase Chain Reaction; TAE, Tris-Acetate-EDTA; HKY, Hasegawa-Kishino-Yano; T92, Tamura 3-parameter; G, gamma; BI, Bayesian Inference; ML, Maximum Likelihood; ABGD, Automatic Barcode Gap Discovery for primary species delimitation; ASAP, Assemble Species by Automatic Partitioning; PTP, Poisson Tree Processes; GMYC, General Mixed Yule Coalescent.
