# Supplementary material for: A Multilocus Integrative Framework to Reassess Species Boundaries Within the Cystoseira Sensu Stricto Complex (Fucales, Phaeophyceae)
Source: Plants (Basel). 2026 Jul 22;15(14):2237. doi: 10.3390/plants15142237 (PMC13415215; doi:10.3390/plants15142237)
Supplement: Supplementary file 1 [file plants-15-02237-s001.zip › plants-4400197-supplementary/Supplementary_rev/File S1 S2 S3.pdf]

**File S1:** sequence clustered in the *cox1* phylogenetic tree

*C. foeniculacea* 1: OK480247, OK480248

*C. foeniculacea* 2: OK480238, OK480239, OK480240, OK480242, OK480249, OK480250, OK480251, OK480252

*C. foeniculacea* 3: 4s1, 4s2, 4s4, 4s8, hr8

*C. foeniculacea* 4: Lan05, Lan07, Lan25, Lan26, Lan27, Lan28

*C. foeniculacea* 5: OK480243, OK480244, OK480245, OK480246

*C. foeniculacea* 6: 2s3, 2s5, 2s7, 3s1, 3s2, 3s10

*C. foeniculacea* 7: 2s2, 2s4, 2s6, 2s8, 2s9

*C. foeniculacea* 8: 2s1

*C. compressa* var. *compressa* 1: 9s1, 9s2, 9s3, 9s7, 10s7

*C. compressa* var. *compressa* 2: OK480266, 9s5, 10s1, 10s3, 10s4, 10s9, 9s5

*C. compressa* var. *compressa* 3: OK480263

*C. compressa* var. *compressa* 4: OK480258, OK480259, OK480260, OK480262, OK480264, OK480265, cad1, cad2, cad3, cad4, cad5

*C. compressa* var. *compressa* 5: OK480253, OK480254, OK480255, OK480256, OK480257, Lan10, Lan23, 3apn, pna

*C. compressa* var. *compressa* 6: dc3p

*C. compressa* var. *compressa* 7: hr5

*C. compressa* var. *compressa* 8: OK480273

*C. compressa* var. *compressa* 9: OK480267, OK480268, OK480269, OK480270, OK480271, OK480272, 8s2, 8s4, 8s6, 8s7, 8s9, 8s10

*C. compressa* var. *pustulata* 1: OK480320, OK480321, OK480322, OK480323, OK480324, hr4, hr7, 1s2, 1s4, 1s6, 1s9, 1s10, 11s1, 11s2, 11s3, 11s5, 11s9, 11s10, 15s2, 15s6, 15s8, 15s10, 7mc, grec1, grec3, grec5, grec9

*C. compressa* var. *pustulata* 2: mch1, mch3mch5, mch6

*C. compressa* var. *pustulata* 3: OK480305, OK480306, OK480307, OK480309, OK480310, OK480311, OK480312, OK480314, OK480315

*C. compressa* var. *pustulata* 4: OK480303, OK480304, OK480308, OK480316, OK480317, OK480318, OK480319, Lan04, Lan29, Lan33, Lan44

*C.* morphotype *canariensis* 1: OK480276, OK480277, OK480278, OK480279, OK480280, OK480282, OK480283, OK480284, OK480285, OK480286, OK480287, OK480288, OK480289, OK480290, OK480296, Marh7, Marh10

*C.* morphotype *canariensis* 2: OK480274, OK480275

*C.* morphotype *canariensis* 3: OK480301, OK480302

*C.* morphotype *canariensis* 4: OK480298, OK480299

*C.* morphotype *canariensis* 5: OK480297

*C.* morphotype *canariensis* 6: OK480291, OK480292

*C.* morphotype *canariensis* 7: Lan42a, Lan42b

*C.* morphotype *canariensis* 8: OK480293, OK480294, OK480295, OK480300, Lan08, Lan11, Lan43

**File S2:** sequence clustered in the *rbcL-rbcS* phylogenetic tree

*C. foeniculacea* 1: mcf2

*C. foeniculacea* 2: 2s4, 2s7, 2s8, 2s9, 3s4, 3s5, 3s7, 3s8, 3s9, 4s1, 4s2, 4s3, 4s4, 4s8, 14s1, 14s2, 14s4, 14s6, 14s9, hr8, 9mc, Lan07, Lan27, Lan28

*C. compressa* var. *pustulata* 1: Lan44

*C. compressa* var. *pustulata* 2: mch3, mch9, 1s4, 1s6, 1s9, 1s10, 11s5, 15s2, 15s5, 15s6, 15s8, 15s10

*C. compressa* var. *compressa* 1: 9s5, 10s1, 10s9

*C. compressa* var. *compressa* 2: 1apn, Lan10, Lan23, 8s2, 8s10, 9s1, 9s2, 9s3, 9s7, 10s7

*C.* morphotype *canariensis* 1: Lan11

*C.* morphotype *canariensis* 2: Marh7, Marh10

*C.* morphotype *canariensis* 3: Marh8, Marh9

**File S3:** sequence clustered in the *ITS2* phylogenetic tree

*C. foeniculacea*: 2s4, 2s5, 2s7, 2s8, 2s9, 3s4, 3s5, 3s7, 3s8, 3s9, 4s1, 4s2, 4s3, 4s4, 14s1, 14s2, 14s4, 14s6, 14s9, hr6, Lan05, Lan07, Lan25, Lan27, Lan28, Lan40, Lan41, mcf2, mcf4, mcf7, mcf8, mcf9, strf1, strf4, strf6, 9mc, dc1p

*C. compressa*: 1s4, 1s6, 1s9, 1s10, 8s2, 8s4, 8s8, 8s10, 9s1, 9s3, 9s4, 9s5, 9s7, 10s1, 10s3, 10s4, 10s7, 10s9, 11s1, 11s5, 11s8, 11s9, 11s10, 15s2, 15s5, 15s6, 15s8, 15s10, Lan10, Lan11, Lan23, Lan29, Lan33, Lan42a, Lan42b, Lan43, Lan44, 1apn, 3apn, cad1, cad2, cad3, cad4, cad5, hr4, hr7, mch1, mch3, mch5, mch6, mch9, pna, 7mc, grec1, grec3, grec5, grec7, grec9

*C.* morphotype *canariensis*: Marh7, Marh8, Marh9, Marh10
