# Supplementary material for: A Multilocus Integrative Framework to Reassess Species Boundaries Within the Cystoseira Sensu Stricto Complex (Fucales, Phaeophyceae)
Source: Plants (Basel). 2026 Jul 22;15(14):2237. doi: 10.3390/plants15142237 (PMC13415215; doi:10.3390/plants15142237)
Supplement: Supplementary file 1 [file plants-15-02237-s001.zip › plants-4400197-supplementary/Supplementary_rev/Supplementary Figures Captions.pdf]

Supplementary Figures' captions:

Figure S1: Sampling sites across the Atlantic Ocean (panel A) and Mediterranean Sea (panel B).

Figure S2: Multilocus Bayesian Inference (BI) and Maximum Likelihood (ML) consensus tree based on the concatenated dataset (at lea*ITS2* + *rbcL–rbcS* + *cox1*) with missing data for *Cystoseira* s.s.. Numbers near nodes indicate Bayesian posterior probabilities (left) and ML bootstrap support values (right).
