# Supplementary material for: A Multilocus Integrative Framework to Reassess Species Boundaries Within the Cystoseira Sensu Stricto Complex (Fucales, Phaeophyceae)
Source: Plants (Basel). 2026 Jul 22;15(14):2237. doi: 10.3390/plants15142237 (PMC13415215; doi:10.3390/plants15142237)
Supplement: Supplementary file 1 [file plants-15-02237-s001.zip › plants-4400197-supplementary/Supplementary_rev/Table S1.pdf]

Tab S1: List of samples used for phylogenetic and morphologic analyses

Species	Sample_ID	Origin	Coordinates	Genbank accession numbers		
				COI	ITS	RBC
Cystoseira compressa var. *pustulata*	Lan04	Spain, Canary Islands, Lanzarote	29º10'48.17" N, 13º25'16.08" W	PX501921		
Cystoseira foeniculacea	Lan05	Spain, Canary Islands, Lanzarote	29º10'48.17" N, 13º25'16.08" W	PX501884	PX639424	
Cystoseira foeniculacea	Lan07	Spain, Canary Islands, Lanzarote	29º10'48.17" N, 13º25'16.08" W	PX501885	PX639425	PX501947
Cystoseira morphotype *canariensis*	Lan08	Spain, Canary Islands, Lanzarote	29º10'48.17" N, 13º25'16.08" W	PX501891		
Cystoseira compressa var. *compressa*	Lan10	Spain, Canary Islands, Lanzarote	28º53'27.85" N, 13º52'34.67" W	PX501864	PX639394	PX501942
Cystoseira morphotype *canariensis*	Lan11	Spain, Canary Islands, Lanzarote	28º53'27.85" N, 13º52'34.67" W	PX501892	PX639434	PX501981
Cystoseira compressa var. *compressa*	Lan23	Spain, Canary Islands, Lanzarote	28º58'52.55" N, 13º49'56" W	PX501865	PX639395	PX501943
Cystoseira foeniculacea	Lan25	Spain, Canary Islands, Lanzarote	29º9'12.9" N, 13º25'45.3" W	PX501886	PX639419	
Cystoseira foeniculacea	Lan26	Spain, Canary Islands, Lanzarote	29º9'12.9" N, 13º25'45.3" W	PX501887		
Cystoseira foeniculacea	Lan27	Spain, Canary Islands, Lanzarote	29º9'12.9" N, 13º25'45.3" W	PX501888	PX639420	PX501944
Cystoseira foeniculacea	Lan28	Spain, Canary Islands, Lanzarote	29º9'12.9" N, 13º25'45.3" W	PX501889	PX639421	PX501945
Cystoseira compressa var. *pustulata*	Lan29	Spain, Canary Islands, Lanzarote	29º9'12.9" N, 13º25'45.3" W	PX501922	PX639396	
Cystoseira compressa var. *pustulata*	Lan33	Spain, Canary Islands, Lanzarote	29º7'11.9" N, 13º38'21.39" W	PX501923	PX639397	
Cystoseira foeniculacea	Lan40	Spain, Canary Islands, Lanzarote	29º6'41.9" N, 13º39'48.86" W		PX639422	
Cystoseira foeniculacea	Lan41	Spain, Canary Islands, Lanzarote	29º6'41.9" N, 13º39'48.86" W		PX639423	
Cystoseira morphotype *canariensis*	Lan42a	Spain, Canary Islands, Lanzarote	29º6'41.9" N, 13º39'48.86" W	PX501893	PX639435	
Cystoseira morphotype *canariensis*	Lan42b	Spain, Canary Islands, Lanzarote	29º6'41.9" N, 13º39'48.86" W	PX501894	PX639436	
Cystoseira morphotype *canariensis*	Lan43	Spain, Canary Islands, Lanzarote	29º6'41.9" N, 13º39'48.86" W	PX501895	PX639437	
Cystoseira compressa var. *pustulata*	Lan44	Spain, Canary Islands, Lanzarote	29º17'6.17" N, 13º29'53.23" W	PX501924	PX639476	PX501946
Cystoseira compressa var. *compressa*	pna	Spain, Canary Islands, Tenerife	28°12'0.05" N, 16°49'41.37" W	PX501925	PX639458	
Cystoseira compressa var. *compressa*	1apn	Spain, Canary Islands, Tenerife	28°12'0.05" N, 16°49'41.37" W		PX639443	PX501971
Cystoseira compressa var. *compressa*	3apn	Spain, Canary Islands, Tenerife	28°12'0.05" N, 16°49'41.37" W	PX501913	PX639444	
Cystoseira compressa var. *pustulata*	7mc	Italy, Cilento, Isola Licosa	40°15'12.0"N, 14°53'56.1"E	PX501914	PX639469	
Cystoseira foeniculacea	9mc	Italy, Cilento, Isola Licosa	40°15'12.0"N, 14°53'56.1"E		PX639445	PX501966
Cystoseira foeniculacea	dc1p	Italy, Cilento, Isola Licosa	40°15'12.0"N, 14°53'56.1"E		PX639451	
Cystoseira compressa var. *compressa*	dc3p	Italy, Cilento, Isola Licosa	40°15'12.0"N, 14°53'56.1"E	PX501915		
Cystoseira morphotype *canariensis*	marh7	Morocco, El Jadida	33°14'40.9" N, 8°32'39.5" W	PX501897	PX639439	PX501977

Cystoseira morphotype *canariensis*	marh8	Morocco, El Jadida	33°14'40.9" N, 8°32'39.5" W		PX639440	PX501980
Cystoseira morphotype *canariensis*	marh9	Morocco, El Jadida	33°14'40.9" N, 8°32'39.5" W		PX639441	PX501978
Cystoseira morphotype *canariensis*	marh10	Morocco, El Jadida	33°14'40.9" N, 8°32'39.5" W	PX501896	PX639438	PX501979
Cystoseira compressa var. *pustulata*	1s2	Italy, Sicilia, Porto Palo di Capo Passero	36°39'6.09 N, 15°4'38.73" E	PX501909		
Cystoseira compressa var. *pustulata*	1s4	Italy, Sicilia, Porto Palo di Capo Passero	36°39'6.09 N, 15°4'38.73" E	PX501910	PX639382	PX501961
Cystoseira compressa var. *pustulata*	1s6	Italy, Sicilia, Porto Palo di Capo Passero	36°39'6.09 N, 15°4'38.73" E	PX501911	PX639383	PX501960
Cystoseira compressa var. *pustulata*	1s9	Italy, Sicilia, Porto Palo di Capo Passero	36°39'6.09 N, 15°4'38.73" E	PX501912	PX639384	PX501956
Cystoseira compressa var. *pustulata*	1s10	Italy, Sicilia, Porto Palo di Capo Passero	36°39'6.09 N, 15°4'38.73" E	PX501908	PX639381	PX501955
Cystoseira foeniculacea	2s1	Italy, Sicilia, Porto Palo di Capo Passero	36°39'6.09 N, 15°4'38.73" E	PX501867		
Cystoseira foeniculacea	2s2	Italy, Sicilia, Porto Palo di Capo Passero	36°39'6.09 N, 15°4'38.73" E	PX501868		
Cystoseira foeniculacea	2s3	Italy, Sicilia, Porto Palo di Capo Passero	36°39'6.09 N, 15°4'38.73" E	PX501869		
Cystoseira foeniculacea	2s4	Italy, Sicilia, Porto Palo di Capo Passero	36°39'6.09 N, 15°4'38.73" E	PX501870	PX639403	PX501932
Cystoseira foeniculacea	2s5	Italy, Sicilia, Porto Palo di Capo Passero	36°39'6.09 N, 15°4'38.73" E	PX501871	PX639404	
Cystoseira foeniculacea	2s6	Italy, Sicilia, Porto Palo di Capo Passero	36°39'6.09 N, 15°4'38.73" E	PX501872		
Cystoseira foeniculacea	2s7	Italy, Sicilia, Porto Palo di Capo Passero	36°39'6.09 N, 15°4'38.73" E	PX501873	PX639405	PX501933
Cystoseira foeniculacea	2s8	Italy, Sicilia, Porto Palo di Capo Passero	36°39'6.09 N, 15°4'38.73" E	PX501874	PX639406	PX501934
Cystoseira foeniculacea	2s9	Italy, Sicilia, Porto Palo di Capo Passero	36°39'6.09 N, 15°4'38.73" E	PX501875	PX639407	PX501935
Cystoseira foeniculacea	3s1	Italy, Sicilia, Porto Palo di Capo Passero	36°39'6.09 N, 15°4'38.73" E	PX501876		
Cystoseira foeniculacea	3s2	Italy, Sicilia, Porto Palo di Capo Passero	36°39'6.09 N, 15°4'38.73" E	PX501878		
Cystoseira foeniculacea	3s4	Italy, Sicilia, Porto Palo di Capo Passero	36°39'6.09 N, 15°4'38.73" E		PX639408	PX501936

Cystoseira foeniculacea	3s5	Italy, Sicilia, Porto Palo di Capo Passero	36°39'6.09 N, 15°4'38.73" E		PX639409	PX501937
Cystoseira foeniculacea	3s7	Italy, Sicilia, Porto Palo di Capo Passero	36°39'6.09 N, 15°4'38.73" E		PX639410	PX501938
Cystoseira foeniculacea	3s8	Italy, Sicilia, Porto Palo di Capo Passero	36°39'6.09 N, 15°4'38.73" E		PX639412	PX501939
Cystoseira foeniculacea	3s9	Italy, Sicilia, Porto Palo di Capo Passero	36°39'6.09 N, 15°4'38.73" E		PX639413	PX501940
Cystoseira foeniculacea	3s10	Italy, Sicilia, Porto Palo di Capo Passero	36°39'6.09 N, 15°4'38.73" E	PX501877		
Cystoseira foeniculacea	4s1	Italy, Sicilia, Porto Palo di Capo Passero	36°39'6.09 N, 15°4'38.73" E	PX501879	PX639414	PX501957
Cystoseira foeniculacea	4s2	Italy, Sicilia, Porto Palo di Capo Passero	36°39'6.09 N, 15°4'38.73" E	PX501880	PX639415	PX501962
Cystoseira foeniculacea	4s3	Italy, Sicilia, Porto Palo di Capo Passero	36°39'6.09 N, 15°4'38.73" E		PX639416	PX501963
Cystoseira foeniculacea	4s4	Italy, Sicilia, Porto Palo di Capo Passero	36°39'6.09 N, 15°4'38.73" E	PX501881	PX639417	PX501964
Cystoseira foeniculacea	4s8	Italy, Sicilia, Porto Palo di Capo Passero	36°39'6.09 N, 15°4'38.73" E	PX501882		PX501965
Cystoseira compressa var. *compressa*	8s2	Italy, Sicilia, Marzamemi	36°44'37.70" N, 15°7'7.41" E	PX501853	PX639386	PX501941
Cystoseira compressa var. *compressa*	8s4	Italy, Sicilia, Marzamemi	36°44'37.70" N, 15°7'7.41" E	PX501854	PX639387	
Cystoseira compressa var. *compressa*	8s6	Italy, Sicilia, Marzamemi	36°44'37.70" N, 15°7'7.41" E	PX501855		
Cystoseira compressa var. *compressa*	8s7	Italy, Sicilia, Marzamemi	36°44'37.70" N, 15°7'7.41" E	PX501856		
Cystoseira compressa var. *compressa*	8s8	Italy, Sicilia, Marzamemi	36°44'37.70" N, 15°7'7.41" E		PX639388	
Cystoseira compressa var. *compressa*	8s9	Italy, Sicilia, Marzamemi	36°44'37.70" N, 15°7'7.41" E	PX501857		
Cystoseira compressa var. *compressa*	8s10	Italy, Sicilia, Marzamemi	36°44'37.70" N, 15°7'7.41" E	PX501852	PX639385	PX501958
Cystoseira compressa var. *compressa*	9s1	Italy, Sicilia, Priolo Gargallo	37°08'45.1"N 15°14'35.2"E	PX501858	PX639389	PX501926
Cystoseira compressa var. *compressa*	9s2	Italy, Sicilia, Priolo Gargallo	37°08'45.1"N 15°14'35.2"E	PX501859		PX501927
Cystoseira compressa var. *compressa*	9s3	Italy, Sicilia, Priolo Gargallo	37°08'45.1"N 15°14'35.2"E	PX501860	PX639390	PX501928
Cystoseira compressa var. *compressa*	9s4	Italy, Sicilia, Priolo Gargallo	37°08'45.1"N 15°14'35.2"E		PX639391	
Cystoseira compressa var. *compressa*	9s5	Italy, Sicilia, Priolo Gargallo	37°08'45.1"N 15°14'35.2"E	PX501861	PX639392	PX501959
Cystoseira compressa var. *compressa*	9s7	Italy, Sicilia, Priolo Gargallo	37°08'45.1"N 15°14'35.2"E	PX501862	PX639393	PX501972
Cystoseira compressa var. *compressa*	10s1	Italy, Sicilia, Priolo Gargallo	37°08'45.1"N 15°14'35.2"E	PX501847	PX639411	PX501929
Cystoseira compressa var. *compressa*	10s3	Italy, Sicilia, Priolo Gargallo	37°08'45.1"N 15°14'35.2"E	PX501848	PX639378	

Cystoseira compressa var. *compressa*	10s4	Italy, Sicilia, Priolo Gargallo	37°08'45.1"N 15°14'35.2"E	PX501849	PX639379	
Cystoseira compressa var. *compressa*	10s7	Italy, Sicilia, Priolo Gargallo	37°08'45.1"N 15°14'35.2"E	PX501850	PX639380	PX501930
Cystoseira compressa var. *compressa*	10s9	Italy, Sicilia, Priolo Gargallo	37°08'45.1"N 15°14'35.2"E	PX501851	PX639442	PX501968
Cystoseira compressa var. *pustulata*	11s1	Italy, Sicilia, Marzamemi	36°44'37.70" N, 15°7'7.41" E	PX501898	PX639459	
Cystoseira compressa var. *pustulata*	11s2	Italy, Sicilia, Marzamemi	36°44'37.70" N, 15°7'7.41" E	PX501900		
Cystoseira compressa var. *pustulata*	11s3	Italy, Sicilia, Marzamemi	36°44'37.70" N, 15°7'7.41" E	PX501901		
Cystoseira compressa var. *pustulata*	11s5	Italy, Sicilia, Marzamemi	36°44'37.70" N, 15°7'7.41" E	PX501902	PX639461	PX501931
Cystoseira compressa var. *pustulata*	11s8	Italy, Sicilia, Marzamemi	36°44'37.70" N, 15°7'7.41" E		PX639462	
Cystoseira compressa var. *pustulata*	11s9	Italy, Sicilia, Marzamemi	36°44'37.70" N, 15°7'7.41" E	PX501903	PX639463	
Cystoseira compressa var. *pustulata*	11s10	Italy, Sicilia, Marzamemi	36°44'37.70" N, 15°7'7.41" E	PX501899	PX639460	
Cystoseira foeniculacea	14s1	Italy, Sicilia, Vendicari	36°48'9.69" N, 15°5'59.92" E		PX639398	PX501967
Cystoseira foeniculacea	14s2	Italy, Sicilia, Vendicari	36°48'9.69" N, 15°5'59.92" E		PX639399	PX501969
Cystoseira foeniculacea	14s4	Italy, Sicilia, Vendicari	36°48'9.69" N, 15°5'59.92" E		PX639400	PX501948
Cystoseira foeniculacea	14s6	Italy, Sicilia, Vendicari	36°48'9.69" N, 15°5'59.92" E		PX639401	PX501970
Cystoseira foeniculacea	14s9	Italy, Sicilia, Vendicari	36°48'9.69" N, 15°5'59.92" E		PX639402	PX501949
Cystoseira compressa var. *pustulata*	15s2	Italy, Sicilia, Vendicari	36°48'9.69" N, 15°5'59.92" E	PX501905	PX639465	PX501951
Cystoseira compressa var. *pustulata*	15s5	Italy, Sicilia, Vendicari	36°48'9.69" N, 15°5'59.92" E		PX639466	PX501952
Cystoseira compressa var. *pustulata*	15s6	Italy, Sicilia, Vendicari	36°48'9.69" N, 15°5'59.92" E	PX501906	PX639467	PX501953
Cystoseira compressa var. *pustulata*	15s8	Italy, Sicilia, Vendicari	36°48'9.69" N, 15°5'59.92" E	PX501907	PX639468	PX501954
Cystoseira compressa var. *pustulata*	15s10	Italy, Sicilia, Vendicari	36°48'9.69" N, 15°5'59.92" E	PX501904	PX639464	PX501950
Cystoseira foeniculacea	strf1	Slovenia, Strunjan National Park	45°32'22.61" N, 13°36'57.17" E		PX639431	
Cystoseira foeniculacea	strf4	Slovenia, Strunjan National Park	45°32'22.61" N, 13°36'57.17" E		PX639432	
Cystoseira foeniculacea	strf6	Slovenia, Strunjan National Park	45°32'22.61" N, 13°36'57.17" E		PX639433	
Cystoseira compressa var. *pustulata*	grec1	Greece, Euboea, Kallianoi	38°8'26.08" N, 24°28'39.36" E	PX501916	PX639470	
Cystoseira compressa var. *pustulata*	grec3	Greece, Euboea, Kallianoi	38°8'26.08" N, 24°28'39.36" E	PX501917	PX639471	
Cystoseira compressa var. *pustulata*	grec5	Greece, Euboea, Kallianoi	38°8'26.08" N, 24°28'39.36" E	PX501918	PX639472	
Cystoseira compressa var. *pustulata*	grec7	Greece, Euboea, Kallianoi	38°8'26.08" N, 24°28'39.36" E		PX639473	
Cystoseira compressa var. *pustulata*	grec9	Greece, Euboea, Kallianoi	38°8'26.08" N, 24°28'39.36" E	PX501919	PX639474	
Cystoseira compressa var. *pustulata*	mch1	Italy, Sicilia, Isola delle Femmine	38°12'12.52" N, 13°14'29.95" E	PX501843	PX639453	
Cystoseira compressa var. *pustulata*	mch3	Italy, Sicilia, Isola delle Femmine	38°12'12.52" N, 13°14'29.95" E	PX501844	PX639454	PX501975
Cystoseira compressa var. *pustulata*	mch5	Italy, Sicilia, Isola delle Femmine	38°12'12.52" N, 13°14'29.95" E	PX501845	PX639455	
Cystoseira compressa var. *pustulata*	mch6	Italy, Sicilia, Isola delle Femmine	38°12'12.52" N, 13°14'29.95" E	PX501846	PX639456	
Cystoseira compressa var. *pustulata*	mch9	Italy, Sicilia, Isola delle Femmine	38°12'12.52" N, 13°14'29.95" E		PX639457	PX501976

Cystoseira foeniculacea	mcf2	Italy, Sicilia, Isola delle Femmine	38°12'12.52" N, 13°14'29.95" E		PX639426	PX501974
Cystoseira foeniculacea	mcf4	Italy, Sicilia, Isola delle Femmine	38°12'12.52" N, 13°14'29.95" E		PX639427	
Cystoseira foeniculacea	mcf7	Italy, Sicilia, Isola delle Femmine	38°12'12.52" N, 13°14'29.95" E		PX639428	
Cystoseira foeniculacea	mcf8	Italy, Sicilia, Isola delle Femmine	38°12'12.52" N, 13°14'29.95" E		PX639429	
Cystoseira foeniculacea	mcf9	Italy, Sicilia, Isola delle Femmine	38°12'12.52" N, 13°14'29.95" E		PX639430	
Cystoseira compressa var. *pustulata*	hr4	Croatia, Funtana	45°11'11.8" N, 13°35'20.5" E	PX501920	PX639475	
Cystoseira compressa var. *compressa*	hr5	Croatia, Funtana	45°11'52.3" N, 13°34'47.8" E	PX501863		
Cystoseira foeniculacea	hr6	Croatia, Funtana	45°11'58.2" N, 13°35'10.1" E		PX639418	
Cystoseira compressa var. *pustulata*	hr7	Croatia, Premantura	44°46'07.2" N, 13°55'35.1" E	PX501890	PX639452	
Cystoseira foeniculacea	hr8	Croatia, Funtana	45°11'11.8" N, 13°35'20.5" E	PX501883		PX50197
Cystoseira compressa var. *compressa*	cad1	Spain, Cadiz	36°04'20.4"N, 5°25'46.7"W	PX501838	PX639446	
Cystoseira compressa var. *compressa*	cad2	Spain, Cadiz	36°04'20.4"N, 5°25'46.7"W	PX501839	PX639447	
Cystoseira compressa var. *compressa*	cad3	Spain, Cadiz	36°04'20.4"N, 5°25'46.7"W	PX501840	PX639448	
Cystoseira compressa var. *compressa*	cad4	Spain, Cadiz	36°04'20.4"N, 5°25'46.7"W	PX501841	PX639449	
Cystoseira compressa var. *compressa*	cad5	Spain, Cadiz	36°04'20.4"N, 5°25'46.7"W	PX501842	PX639450	
