# Supplementary material for: A Multilocus Integrative Framework to Reassess Species Boundaries Within the Cystoseira Sensu Stricto Complex (Fucales, Phaeophyceae)
Source: Plants (Basel). 2026 Jul 22;15(14):2237. doi: 10.3390/plants15142237 (PMC13415215; doi:10.3390/plants15142237)
Supplement: Supplementary file 1 [file plants-15-02237-s001.zip › plants-4400197-supplementary/Supplementary_rev/Table S2.pdf]

Tab S2: List of additional sequences used for the *cox1* phylogenetic tree and species delimitation methods

Cystoseira foeniculacea	OK480238	Spain: Galicia
	OK480239	
	OK480240	Portugal: Algarve, Ria Formosa
	OK480242	Spain: Tarifa area
	OK480243	Spain: Canary Isl, Tenerife
	OK480244	
	OK480245	Spain: Canary Isl, Gran Canaria
	OK480246	Spain: Canary Isl, Lazarote
	OK480247	Spain: Baleares, Cabrera
	OK480248	
	OK480249	
	OK480250	Spain: Baleares, Menorca
	OK480251	Greece: Crete
	OK480252	
Cystoseira compressa	OK480253	Spain: Canary Isl, Tenerife
	OK480254	
	OK480255	Spain: Canary Isl, Gran Canaria
	OK480256	Portugal: Algarve
	OK480257	
	OK480258	Spain: Tarifa area
	OK480259	Morocco: Kaa Asserasse
	OK480260	Morocco: Nador Lagoon
	OK480262	Spain: Almeria
	OK480263	France: Banyuls
	OK480264	

	OK480265	Spain: Baleares, Menorca
	OK480266	Italy: Sicily, Cefalu
	OK480267	Malta
	OK480268	Italy: Numana
	OK480269	
	OK480270	Italy: Vasto
	OK480271	
	OK480272	Greece: Crete
	OK480273	Israel: Haifa
Cystoseira humilis	OK480274	France: Brittany
	OK480275	
	OK480276	Spain: Galicia
	OK480277	
	OK480278	Portugal: Ericeira
	OK480279	
	OK480280	Portugal: SW Coast
	OK480282	Portugal: Algarve
	OK480283	
	OK480284	Spain: Tarifa area
	OK480285	Morocco: El Jadida area
	OK480286	
	OK480287	Morocco: Essaouira
	OK480288	
	OK480289	Mauritania: Nouadhibou area
	OK480291	
	OK480292	Portugal: Madeira
	OK480293	Portugal: Madeira
	OK480294	Spain: Canary Isl, Lazarote
	OK480295	
	OK480296	Spain: Canary Isl, Fuerteventura
	OK480297	

	OK480298	Spain: Canary Isl, La Gomera
	OK480299	Spain: Canary Isl, Hierro
	OK480300	
	OK480301	Spain: Canary Isl, Gran Canaria
	OK480302	Spain: Canary Isl, Tenerife
Cystoseira pustulata	OK480303	Spain: Canary Isl, Gran Canaria
	OK480304	Spain: Canary Isl, Lazarote
	OK480305	Portugal: Azores, Formigas
	OK480306	Portugal: Azores, Santa Maria
	OK480307	Portugal: Azores, Sao Miguel
	OK480308	
	OK480309	Portugal: Azores, Faial
	OK480311	Portugal: Azores, Pico
	OK480312	
	OK480314	Portugal: Azores, S. Jorge
	OK480315	
	OK480316	Portugal: Azores, Corvo
	OK480317	Spain: Almeria
	OK480318	Spain: Baleares, Menorca
	OK480319	Italy: Pantelleria
	OK480320	
	OK480321	Malta
	OK480322	Greece: Mykonos
	OK480323	Greece: Crete
	OK480324	
