# Supplementary material for: A Multilocus Integrative Framework to Reassess Species Boundaries Within the Cystoseira Sensu Stricto Complex (Fucales, Phaeophyceae)
Source: Plants (Basel). 2026 Jul 22;15(14):2237. doi: 10.3390/plants15142237 (PMC13415215; doi:10.3390/plants15142237)
Supplement: Supplementary file 1 [file plants-15-02237-s001.zip › plants-4400197-supplementary/Supplementary_rev/Table S3.pdf]

Table S3: Morphological characters of the three varieties of *Cystoseira compressa*

Character	*C. compressa* var. *compressa*	*C. compressa* var. *pustulata*	*C.* morphotype *canariensis*
**General habit**	Robust, variable in size (from a few cm in exposed sites up to 1 m in sheltered sites).	More slender and delicate; generally, 10–30 cm tall.	Dense turfs, small; typically 5–15 cm.
**Colour**	Yellowish- to dark-brown, non-iridescent.	Yellowish to light brown, non-iridescent.	Yellowish-brown, non-iridescent.
**Holdfast**	Small, compact, discoid holdfast.	Small, compact, discoid holdfast.	Small, discoid holdfast.
**Cauloid**	Short (1–3 cm), cylindrical or slightly flattened; apices smooth and prominent.	Short (1–3 cm), cylindrical; apices smooth and slightly prominent.	Short (1–3 cm), cylindrical; apices smooth and slightly protruding.
**Primary branches**	Flattened in winter, cylindrical in spring–summer but compressed at the base; elongating up to 20–60 cm (up to 1 m in sheltered sites).	Cylindrical, occasionally slightly compressed at the base; branching from the lower half; generally shorter (never exceeding ~10-30 cm).	Cylindrical, never flattened; 4–15 cm long, progressively attenuating; denuded at base with growth; with crypt-bearing pedicels.
**Secondary and higher-order branches**	Slender (1.5–2 mm), cylindrical in spring-summer; alternate, distichous; numerous in spring–summer, scarce in winter.	Slender (1.5–2 mm), cylindrical; simple or sparsely branched; occasionally distichous in a single plane.	Secondaries up to 2–4 cm long, branched once or twice; tertiaries delicate, often curved, distichous near apices.
**Cryptostomata / Crypts**	Abundant, evenly distributed, not or very slightly protruding; usually arranged in two parallel rows in winter.	Abundant, conspicuous, prominent; evenly distributed on all axes; never pedicellate.	Abundant crypts with highly protruding ostioles on secondary branches; numerous cylindrical crypt-bearing pedicels (0.4–0.6 mm long) on primary branches (diagnostic feature).
**Aerocysts**	Usually present; variable in size: from small (3-6 × 1-2 mm) to large (8–10 mm); subterminal, isolated or arranged in series of 2–3.	Usually absent; if present, very small (2-3 mm).	Always absent.
**Receptacles**	Terminal, compact, fusiform to lanceolate-fusiform (1-10 × 0.5-1 mm); simple or bi- to trifurcate; occasionally pedicellate on aerocysts.	Terminal, compact, fusiform (1-2 × 0.5 mm); simple or rarely bifurcate; never pedicellate.	Terminal on distal secondary branches; simple or sparingly branched, slightly tuberculate or fusiform; 3-5 mm long × 0.3-0.5 mm wide.
**Phenology**	Strongly seasonal. Winter: reduced *rosette form* with short, flattened branches and two-row cryptostomata. Spring–summer: erect, cylindrical, highly branched fronds.	No marked seasonal variation; always cylindrical; winter form reduced but never *rosette-shaped*.	No marked seasonal variation.
**Habitat**	Atlantic, Mediterranean; upper infralittoral (0-1 m), exposed and sheltered sites, also in rock pools; size strongly affected by wave exposure.	Atlantic, Mediterranean; Infralittoral rocky substrates (0.5-5 m) and tide pools; generally deeper than var. *compressa* in the Mediterranean, sometimes down to 20-30 m (reported to 40 m).	Atlantic; upper intertidal pools, basaltic rock pools of Canary Islands; in deeper pools, dwarf forms occur. Also recorded from Morocco, where plants are more robustly developed.
