# Supplementary figures and images for: A Multilocus Integrative Framework to Reassess Species Boundaries Within the Cystoseira Sensu Stricto Complex (Fucales, Phaeophyceae)
Source: Plants (Basel). 2026 Jul 22;15(14):2237. doi: 10.3390/plants15142237 (PMC13415215; doi:10.3390/plants15142237)

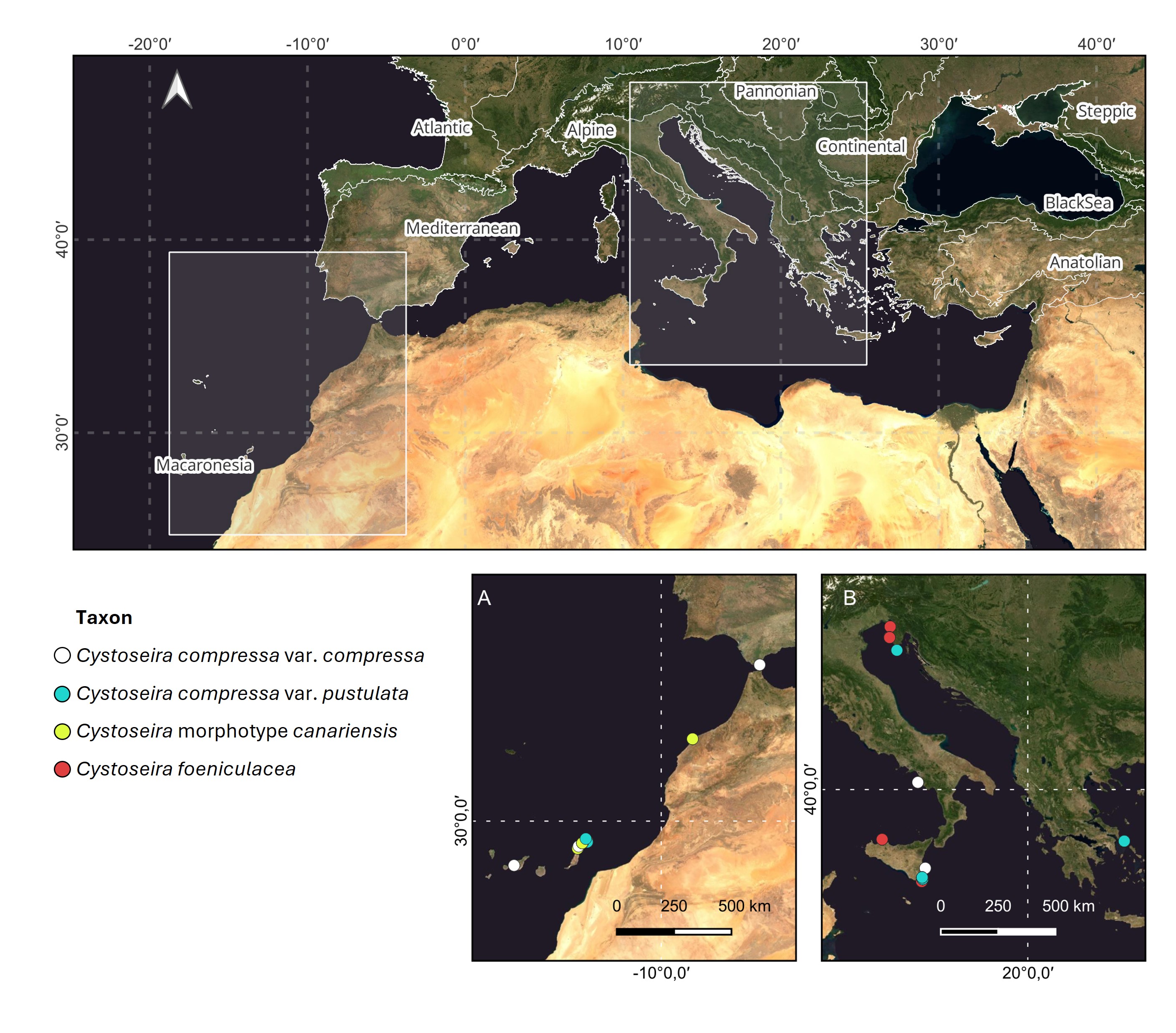

Supplement: Supplementary file 1 [file plants-15-02237-s001.zip › plants-4400197-supplementary/Supplementary_rev/Fig S1 rev.jpg]

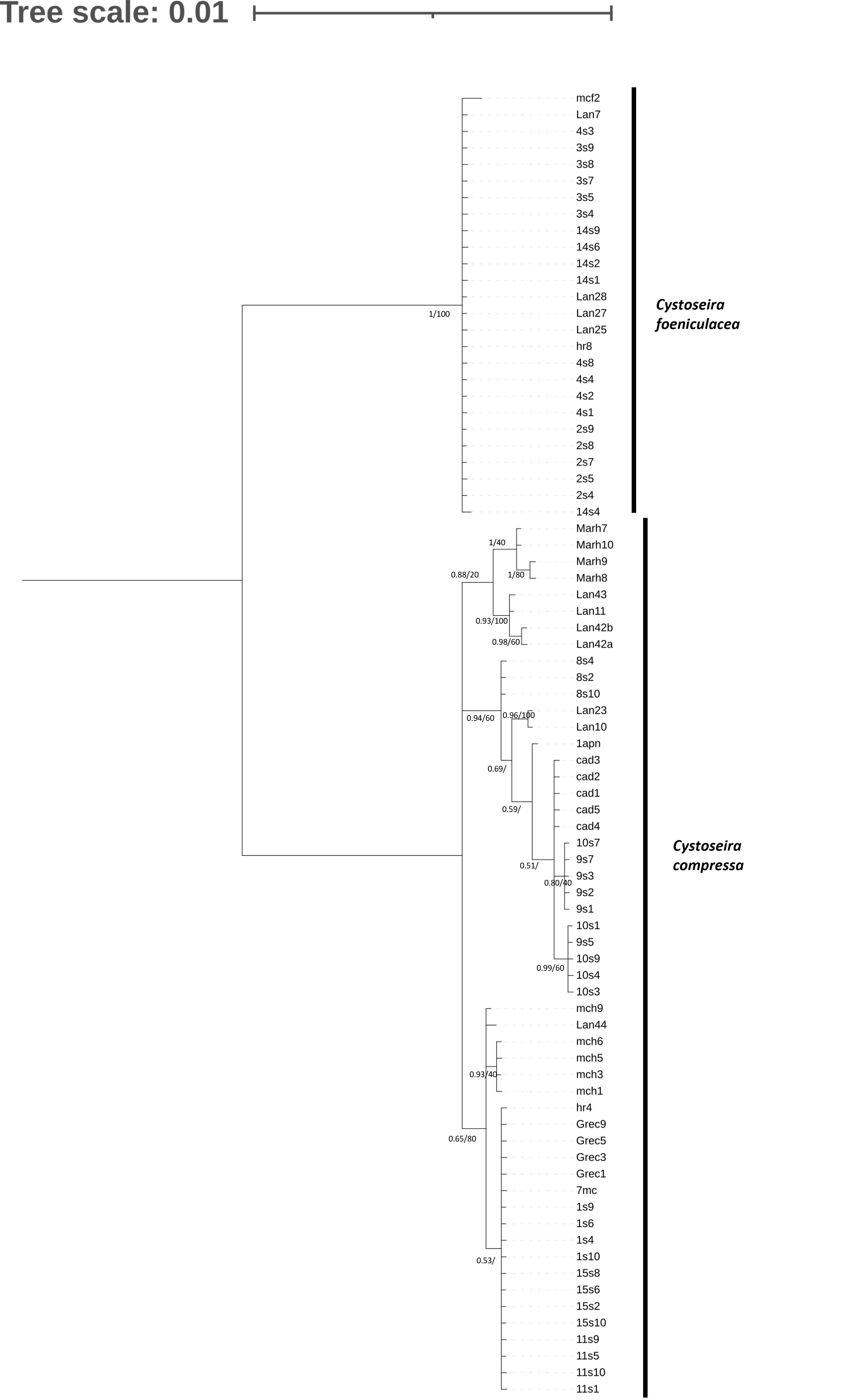

Supplement: Supplementary file 1 [file plants-15-02237-s001.zip › plants-4400197-supplementary/Supplementary_rev/Fig S2.jpg]
